# AI-ECG classification for Brugada syndrome: A study of machine learning techniques to optimise for limited datasets

**DOI:** 10.1371/journal.pdig.0001222

**Published:** 2026-02-25

**Authors:** Keenan Saleh, Raaif Hadadi, Yixiu Liang, Hong Wong, Arunashis Sau, James Howard, Evan Brittain, Jeffrey Annis, Majd El-Harasis, Matthew Shun-Shin, Jagdeep Mohal, Akriti Naraen, Jack Samways, Jessica Artico, James Ware, Prapa Kanagaratnam, Fu Siong Ng, Massoud Zolgharni, Wenjia Bai, Amanda Varnava, Zachary Whinnett, Ahran Arnold

**Affiliations:** 1 National Heart and Lung Institute, Imperial College London, London, United Kingdom of Great Britain & Northern Ireland; 2 Department of Computing, Imperial College London, London, United Kingdom of Great Britain & Northern Ireland; 3 Zhongshan Hospital, Shanghai, China; 4 Vanderbilt University Medical Centre, Nashville, United States of America; 5 Imperial College Healthcare NHS Trust, London, United Kingdom of Great Britain & Northern Ireland; 6 Intelligent Sensing and Vision, University of West London, London, United Kingdom of Great Britain & Northern Ireland; 7 Department of Brain Sciences, Imperial College London, London, United Kingdom of Great Britain & Northern Ireland; University of Tübingen: Eberhard Karls Universitat Tubingen, GERMANY

## Abstract

Deep neural networks can classify ECGs with high accuracy when training data is abundant. Rare conditions like Brugada syndrome, an inherited arrhythmia syndrome predisposing to sudden death, pose challenges due to data scarcity hindering model training. We evaluated multiple machine learning (ML) approaches to optimise a Brugada ECG classification model using limited training data. The baseline model was trained on a dataset comprising 176 Brugada, 176 right bundle branch block (RBBB) and 352 normal ECGs from Zhongshan Hospital (Zhongshan-baseline dataset), framed as a binary classification task to distinguish Brugada from non-Brugada ECGs. A 25%-75% train-test split was used to exacerbate data scarcity. To enhance training, we incorporated three additional datasets: (i) a different, labelled ECG dataset from Zhongshan Hospital including normal and RBBB ECGs (Zhongshan-pretrain), (ii) an unlabelled ECG dataset from Hammersmith Hospital including Brugada and non-Brugada ECGs (Imperial), (iii) an open-access labelled ECG dataset (PTB-XL). Three strategies were tested: (1) supervised pretraining, (2) self-supervised pretraining with data augmentation, and (3) oversampling using SMOTE (synthetic minority oversampling technique). Each model was evaluated on the unseen internal test set and an external Brugada mimic dataset. The models were re-trained using an 80%-20% train-test split as a secondary analysis. The baseline model achieved 92.2% accuracy, F1-score 0.837, and area under the Receiver Operating Characteristic curve (AUC) 0.962. Supervised pretraining significantly improved performance when training data was scarce, with the best model pretrained on the Zhongshan-pretrain dataset boosting accuracy (+3.2%), F1-score (+0.071) and AUC + 0.019), with consistent cross-validation performance. Self-supervised pretraining produced smaller and more variable gains, although select models better mitigated against false positives on the Brugada mimic dataset. SMOTE oversampling showed inconsistent effects on performance. Incorporating pretraining and oversampling may facilitate the development of more accurate AI-ECG models for rare diseases when training data is limited but provides diminishing returns when adequate labelled data is available.

## Author summary

AI applied to ECG interpretation (AI-ECG) is an emerging tool in the field of cardiac diagnostics that can rapidly automate ECG analysis, improving clinical resource utilisation and efficiency. However, rare conditions have limited available data to train AI-ECG models, hindering their performance. In this study, we developed a baseline AI-ECG model for Brugada syndrome using a severely restricted dataset and investigated three strategies to address data scarcity: supervised pretraining, self-supervised pretraining and oversampling. Pretraining involves training the model on a broader dataset before refining it for Brugada classification. This can be supervised, where ECG diagnoses or labels are known to the model, or self-supervised, where the model must learn patterns autonomously without labelled ECG data. Oversampling generates synthetic ECGs to supplement model training. Our results indicate that configurations of each approach provided incremental improvements in model performance and could be applied to the development of AI-ECG models for other rare cardiac diseases. Notably, we highlight the strongest improvements are achieved using supervised pretraining and the potential value of self-supervised pretraining using unlabelled datasets, reducing reliance on resource-intensive manual labelling. Together, these findings show how data-efficient training strategies can support the development of AI-ECG models for rare cardiac diseases and help ensure that advances in AI-driven healthcare do not exacerbate existing health inequalities.

## Introduction

The field of artificial intelligence (AI) applied to electrocardiograph (ECG) interpretation (AI-ECG) has grown considerably in recent years. Deep learning has enabled computational algorithms to perform rapid, automated ECG classification and prediction tasks. With sufficient training, the accuracy and performance of these specialised algorithms is highly impressive, in some cases even demonstrating superhuman abilities to detect subclinical ECG patterns [1].

A key requirement for the development of accurate AI-ECG models has been the provision of large datasets on which these algorithms can learn complex patterns. This is less easily achieved for rare conditions, where there are relatively few cases to learn from. Recent advances in the field of machine learning have highlighted that techniques such as pretraining and synthetic data generation can substantially improve model performance where labelled data are scarce, with applications spanning both medical and non-medical domains [2,3].

Brugada syndrome is a rare inherited cardiac condition with an estimated prevalence as low as 1 in 5000 worldwide [4]. The diagnosis is made by human expert interpretation of the 12-lead ECG. Its association with increased risk of sudden death underscores the importance of accurate ECG classification. Demographic factors such as gender and ethnic background strongly influence the prevalence and clinical presentation of Brugada syndrome, with a greater predominance among males and South East Asian populations [5,6].

In this study, we evaluated and compared machine learning techniques to enhance model performance for ECG classification with restricted training data, using the case example of Brugada syndrome. We aimed to assess the viability of these approaches and explore their potential application in AI-ECG models for other rare ECG diagnoses.

## Methods

### Study design

This was a comparative study assessing different machine learning methods designed to address scarcity of training data for a Brugada ECG classification task. The aim of the classification task was to detect the binary presence or absence of the characteristic Brugada syndrome ECG pattern from 12-lead ECGs. We assessed whether the following techniques improved model performance: (i) supervised pretraining, (ii) self-supervised pretraining approaches using data augmentation and (iii) SMOTE oversampling. We simulated data scarcity by limiting the training data available for model development (25%-75% train-test split). The performance of the machine learning models was evaluated using accuracy, F1-score and area under the receiver operating characteristic curve (AUC). The developed models do not represent a definitive clinically deployable model for Brugada ECG detection, but rather the focus of the study was to illustrate the comparative utility of different machine learning strategies to improve model performance in the context of a scarce dataset. This study was undertaken with Research Ethics Committee approval (24/NS/0032).

### Datasets

In total, four ECG datasets were utilised for model development:

#### 1. Baseline dataset (Zhongshan-baseline dataset).

The train-test dataset for the baseline machine learning model was a labelled set of 704 natively digital 12-lead ECGs from 704 individual patients extracted from the Zhongshan Hospital database. This comprised 352 normal ECGs, 176 right bundle branch block (RBBB) ECGs, which can mimic the Brugada syndrome ECG pattern, and 176 ECGs from patients demonstrating a true Brugada syndrome ECG pattern. All Brugada ECGs were from patients with confirmed Brugada syndrome, with a previous history of a spontaneous or provoked type 1 Brugada ECG pattern. The case mix of ECGs within this dataset included both type 1 and non-type 1 Brugada pattern ECGs. The presence or absence of Brugada pattern, including the presence or absence of the type 1 pattern, on each ECG was verified by an expert in inherited arrhythmia syndromes.

The normal and RBBB ECGs were pooled as “non-Brugada” cases for the binary classification task. The mean age of these patients was 53.3 ± 17.4 years and 31.5% were female.

For pretraining approaches designed to enhance learning from limited training data by utilising different data, the following datasets were applied:

#### 2. Zhongshan-pretrain dataset.

9,648 normal and 9,824 RBBB 12-lead ECGs (from 19,472 individual patients: i.e., no duplicate patients), extracted from the Zhongshan Hospital database. The mean age of these patients was 57.5 ± 18.5 years and 37.4% were female.

#### 3. PTB-XL dataset.

The publicly available PTB-XL dataset from PhysioNet [7], comprising 21,799 12-lead ECGs from 18,869 patients ECGs are pre-labelled as one of five classes: normal ECG, myocardial infarction, ST/T change, conduction disturbance, or hypertrophy. The mean age of these patients was 59.5 ± 16.8 years and 47.9% were female.

#### 4. Imperial dataset.

717 12-lead ECG recordings from 67 patients with confirmed Brugada syndrome from Hammersmith Hospital. These ECGs were acquired via the Labsystem Pro (Boston Scientific, USA) electrophysiology recording system during invasive electrophysiology study procedures and comprise unlabelled recordings including Brugada and non-Brugada pattern ECGs (as the Brugada ECG pattern can be transient and concealed at rest). The mean age of these patients was 50.1 ± 13.2 years and 52.2% were female.

### ECG data processing

The ECG data underwent additional signal processing steps in preparation for the machine learning pipeline. This included down-sampling of ECG data to a uniform sampling rate of 100Hz and normalising the amplitude of the filtered ECG signal. Individual ECG complexes were extracted using an R-wave peak detection algorithm [8], and then applying a 500ms window either side of the R-wave. The ECG processing steps are detailed in the supplementary methods (S1 Appendix). All ECG processing and model development was undertaken using the Python programming language version 3.9 and TensorFlow library version 2.1.

### Baseline model training

**Fig 1:**
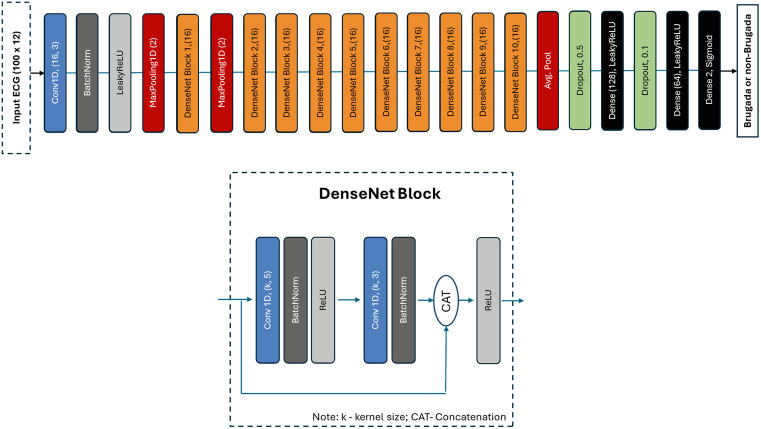
Network architecture. Network architecture featuring a 1D convolutional neural network with DenseNet-style blocks.

Our baseline AI-ECG Brugada classification model was trained to detect the Brugada pattern from 12-lead ECG data. The model architecture consisted of 10 sequential DenseNet blocks as a feature extractor, followed by a fully connected layer classification head (Fig 1). Further details are provided in the supplementary methods (S1 Appendix).

### Train-test split

The primary analysis was undertaken using a stratified 25%-75% train-test split at the patient level, such that a random selection of 25% of the baseline ECGs (Zhongshan-baseline) were partitioned into the training set and the remaining 75% formed the hold-out test set. A secondary analysis was also undertaken using a conventional 80%-20% train-test split for comparison. The dataset was constructed to maintain a ratio of 3:1 of non-Brugada to Brugada ECGs, and this class distribution was maintained across the training and hold-out test sets through stratification. Patient-level grouping was strictly enforced, such that all ECG beats from a given patient were assigned to a single partition and never appeared across training, validation, or test sets. All splits and cross-validation folds were generated using fixed random seeds (random_state = 42) to ensure reproducibility.

### Tested machine learning approaches

Three approaches were applied to determine if they could enhance the baseline model:

1 Supervised pretraining
2 Self-supervised pretraining with data augmentation
3 Oversampling

#### 1. Supervised pretraining.

Our first approach to address the challenges presented by the limited training data was to perform pretraining. This method involves initially training the feature extractor neural network on a larger, more generalised dataset to either perform a separate ECG classification task (supervised learning) or to learn patterns in the data from unlabelled data (self-supervised learning). For supervised pretraining, we trained two separate one-dimensional convolutional neural networks with DenseNet-style blocks, each using a different large, labelled ECG dataset (PTB-XL or Zhongshan-pretrain).

**Fig 2:**
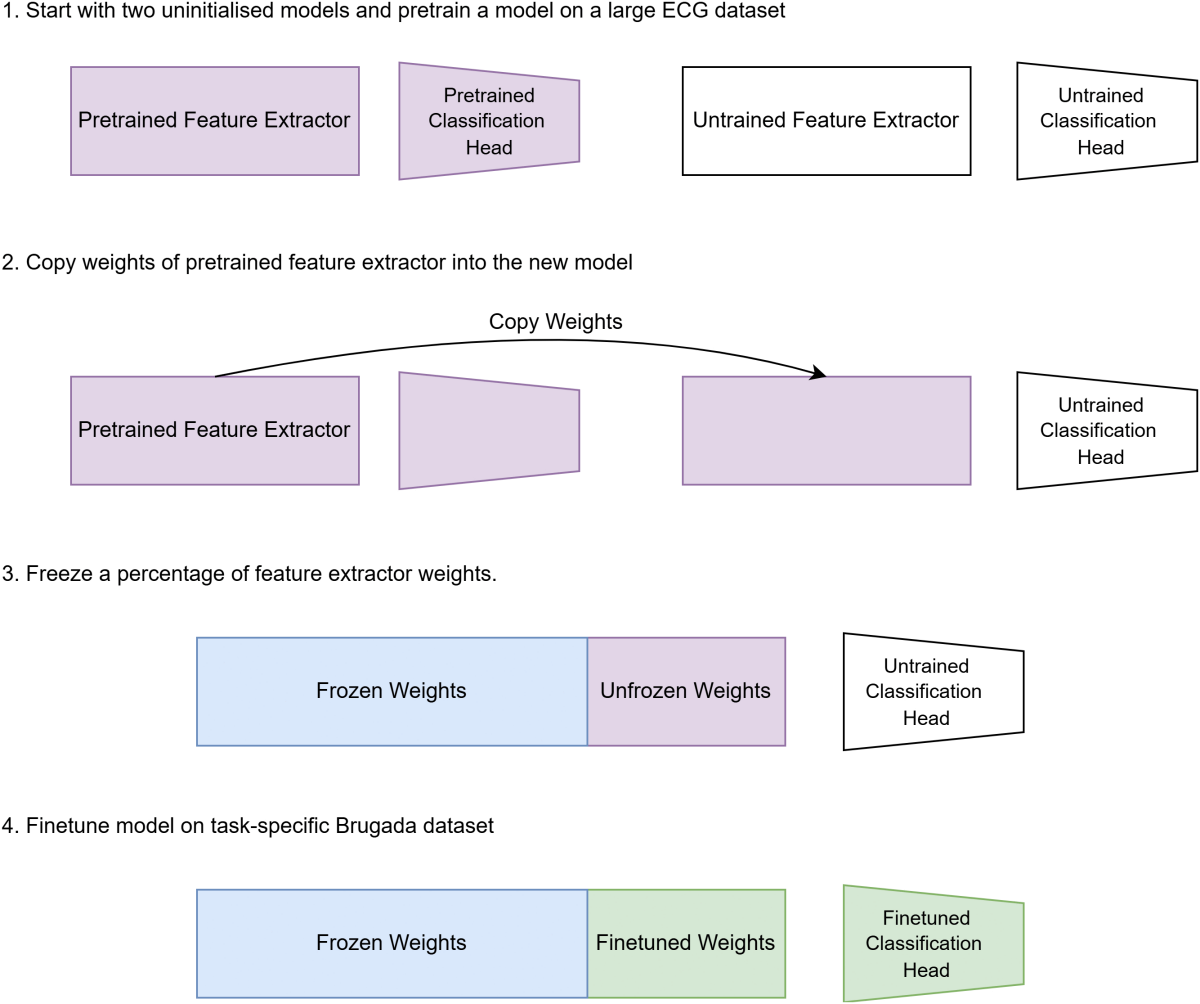
Transfer learning pipeline. Overview of the supervised pretraining and fine-tuning workflow. A convolutional neural network is first pretrained on a large ECG dataset to learn general-purpose feature representations (Step 1). The pretrained feature extractor weights are then transferred to a new task-specific model, while the classification head is reinitialised (Step 2). During fine-tuning, a predefined proportion of the transferred feature extractor layers is frozen to preserve learned representations, while the remaining layers and the classification head are optimised using the Brugada syndrome training dataset (Steps 3–4). This strategy enables knowledge transfer from large-scale ECG data while limiting overfitting in data-scarce settings.

The pretrained model’s knowledge (i.e., its weights) was then transferred into a new fine-tuned model with an identical feature extractor architecture (Fig 2). The majority of the transferred weights are frozen, leaving only the weights of the classification head and the final layers of the feature extractor (specifically the final two DenseNet blocks) to be iteratively trained when applied to the downstream binary classification task of identifying a Brugada ECG from the baseline dataset (Zhongshan-baseline). The resulting lower number of trainable weights enables the model to learn to classify Brugada ECGs from significantly less training data. This approach also helps to overcome overfitting challenges, where model performance on the training dataset does not generalise to the test dataset.

RBBB and ischaemic ST elevation are ECG patterns that can mimic the Brugada pattern. These patterns and true Brugada ECG patterns can be mistaken for each other by human ECG assessors. We hypothesised that a model trained to identify either RBBB, ischaemic ST elevation or other similarly abnormal ECGs would extract similar features and patterns to Brugada ECGs. This would clearly be advantageous in the development of our Brugada ECG detection model, as a greater proportion of model development can be undertaken using easily obtained and/or widely available ECG data.

Two feature extractor models were pretrained on separate supervised classification tasks with pre-labelled ECG data. The first model was trained to distinguish between RBBB and normal ECGs (binary classification), in an approach similar to that taken by Liu et al [9]. This was achieved by training the model on labelled normal and RBBB ECGs from the Zhongshan-pretrain dataset. A second separate model was pretrained using the PTB-XL dataset to classify five different classes of ECG data, enabling a broader range of ECG features to be learnt by the model.

#### 2. Self-supervised pretraining.

**Fig 3:**
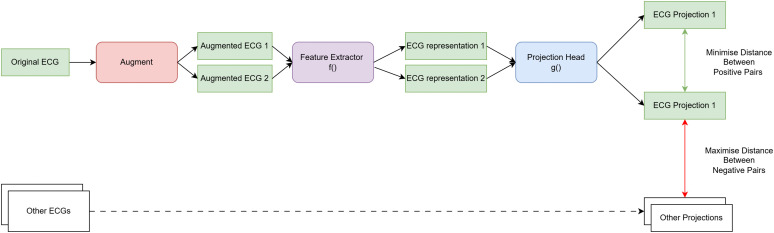
Pair contrastive learning pipeline. Self-supervised contrastive pretraining framework (SimCLR) applied to ECG data. An original ECG is transformed into two augmented views using stochastic signal perturbations. Both augmented signals are passed through a shared feature extractor f(·to produce latent representations, which are further mapped by a projection head g(·) into a contrastive embedding space. The training objective minimises the distance between embeddings derived from different augmentations of the same ECG (positive pairs), while maximising the distance between embeddings from different ECGs within the batch (negative pairs). After pretraining, the projection head is discarded and the feature extractor is used for downstream Brugada classification.

We explored self-supervised learning techniques for pretraining models on ECG data. Instead of directly classifying ECG signals, we leveraged contrastive learning, which focuses on identifying relationships between pairs of ECG signals. By learning to differentiate between pairs of signals that are similar (positive pairs) and those that are dissimilar (negative pairs), the model can develop a robust understanding of the data’s underlying structure and patterns (Fig 3).

As with supervised pretraining, the pretrained model’s weights are transferred to a new fine-tuned model. We applied this approach using two established frameworks:

(i) SimCLR (Simple Framework for Contrastive Learning of Visual Representations) [10]
(ii) MoCo-V2 (Momentum Contrast for Unsupervised Visual Representation Learning v2) [11]

The SimCLR framework uses the same backbone as in Fig 1 with a two-layer ReLU projection head and allows the model to directly learn generalisable features from the augmented ECGs. MoCo-V2 replaces large-batch negatives with a dynamic memory queue of encoded samples, which is more computationally efficient. Both frameworks are well described in machine learning applications for ECG classification [12–14]. Self-supervised pretraining was undertaken using one of three datasets without labels. Either the unlabelled Imperial dataset, or the delabelled PTB-XL or Zhongshan-pretrain datasets.

**Fig 4:**
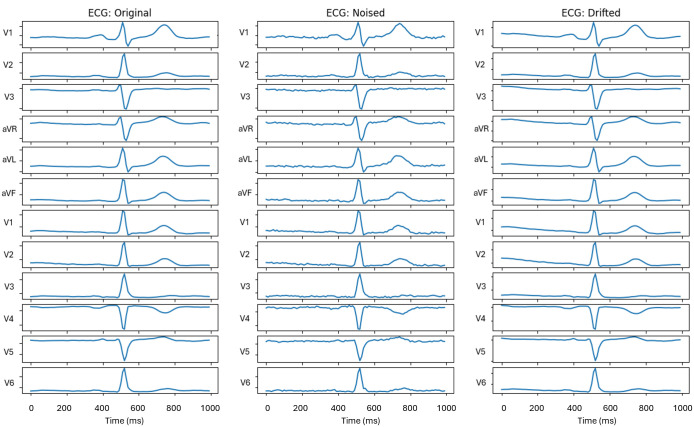
ECG augmentation techniques. Examples of ECG augmentations used during self-supervised contrastive pretraining. Representative 12-lead ECG beat shown in its original form (left), after addition of Gaussian noise (centre), and after addition of baseline drift (right). Each column displays the same cardiac beat across all 12 leads. Augmentations were designed to introduce realistic signal variability while preserving diagnostically relevant morphology. These transformed views were used to generate positive pairs during contrastive pretraining, encouraging the model to learn invariant ECG representations robust to noise and baseline shifts.

Data augmentation is key to the methodology of pair contrastive learning frameworks. This is the artificial generation of new training data by making minor transformations to the original data. Associating augmented ECG signals with their originals yields additional positive pairs which can subsequently be incorporated into the training process, thus improving representation learning and maximising the efficiency of the available training data. To maintain medical accuracy, we focused on data augmentation techniques which reflected real-world ECG variations and that reflect common sources of variability in ECG acquisition. These included the injection of random baseline drift, mimicking respiratory movement, and superimposition of high frequency white Gaussian noise, mimicking fibrillation waves (Fig 4).

Because the effectiveness of data augmentation in contrastive learning is highly domain dependent, we performed a systematic ablation study for each dataset in which individual augmentations were applied during self-supervised pretraining and evaluated using downstream performance. This analysis revealed that while some clinically plausible perturbations can be beneficial, others consistently degrade performance by removing diagnostically relevant morphological information. The results of the ablation study are presented in **Table A in** S1 Appendix. Consequently, augmentation strategies were selected empirically rather than based on assumed physiological realism. These augmentations were applied only to self-supervised pretraining to facilitate contrastive learning. We detail the steps and parameters used for data augmentation within the supplementary methods (S1 Appendix).

#### 3. Oversampling.

Oversampling techniques seek to address class imbalance within the training dataset. This involves the generation of synthetic ECGs modelled on the appearance of ECGs from the minority class (i.e., the Brugada ECGs). These synthetic ECGs preserve important relationships within the data while allowing generalisable features to be learnt and recreated in new artificial examples.

**Fig 5:**
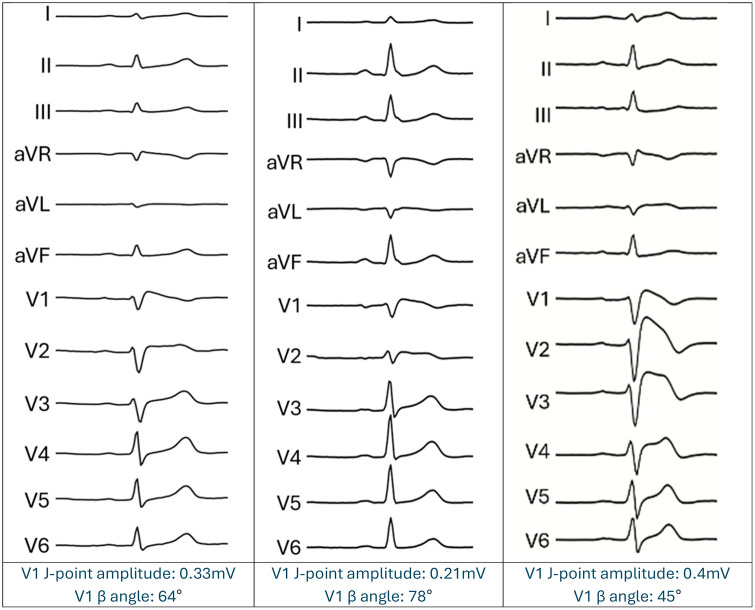
SMOTE-generated Brugada ECGs. Three example SMOTE-generated Brugada ECGs, with associated J-point amplitude and β angle in lead V1.

The synthetic minority oversampling technique (SMOTE) interpolates new data points between the original ECG and its nearest neighbour from the same ECG class, thus producing a new slightly different ECG which is still representative of the underlying condition (Fig 5). SMOTE has been applied to raw ECG signals for data generation and augmentation in other arrhythmia classification models [15,16]. Addressing the class imbalance of Brugada ECGs relative to non-Brugada ECGs in our training dataset may improve our Brugada classification model performance. We have provided example SMOTE-generated Brugada ECGs in Fig 5. We further detail how SMOTE was undertaken within the supplementary methods (S1 Appendix).

All synthetic ECGs were visually reviewed by an expert in inherited arrhythmia syndromes to confirm plausibility. This manual inspection provided basic assurance of waveform validity in the absence of a more objective, recognised method for validating the physiological plausibility of synthetic ECGs.

### Statistical analysis

The performance of the Brugada classification models was evaluated using accuracy, F1-score and AUC (area under the receiver operating characteristic curve), sensitivity, specificity, negative predictive value (NPV), positive predictive value (PPV), averaged precision (area under the precision-recall curve) and Brier score. Differences in AUC were assessed using the DeLong non-parametric test. Paired classification differences at a fixed decision threshold were evaluated using McNemar’s test. A two-sided p-value <0.05 was considered statistically significant, with multiplicity controlled using the Holm step-down procedure. Cross-validation performance is reported as the mean ± standard deviation with 95% confidence intervals across folds. Bootstrap resampling was used to compared selected pretrained models for further analysis. Test set beats were resampled with replacement 1000 times, with identical resampling applied to both models to preserve pairing. For each replicate, all prespecified metrics were recomputed and metric differences recorded. We report the mean difference and 95% bootstrap confidence intervals, with empirical two-sided p-values derived from the bootstrap distribution. Descriptive statistics have also been included where appropriate.

### Brugada mimic stress test

We undertook a supplemental analysis to evaluate the false positive rate of each developed model against a curated dataset of solely “Brugada mimics”, extracted from Vanderbilt University Medical Center. This 611 ECG dataset included RBBB cases (n = 200) and other mimics including early repolarisation (n = 200), anterior ST elevation myocardial infarction (n = 200) and hypertrophic cardiomyopathy (n = 11). The mean age of these patients was 53.6 ± 19.9 years and 24% were female.

### Model explainability

SHapley Additive exPlanations (SHAP) was applied as a post-hoc explainability technique. SHAP GradientExplainer was applied using a randomly selected background set of 100 training samples to serve as the reference distribution for feature attribution. SHAP values were computed for all test samples with Brugada syndrome. SHAP overlays were also generated for a random subset of Brugada samples, showing lead-wise temporal attributions across the ECG waveform. To assess whether these attributions were meaningfully linked to learned model parameters rather than artefacts of the input, we performed the parameter randomisation sanity check proposed by Adebayo et al [17]. For each model, network weights were progressively re-initialised in a top-down cascade while recomputing SHAP attributions, and attribution similarity was quantified using Spearman correlation between the flattened absolute SHAP maps of the trained and randomised models across Brugada ECG test samples.

## Results

Test set performance for the 25%-75% data split is shown in Table 1, with cross-validation results provided in Table B in S1 Appendix. The baseline model performance, without the implementation of additional pretraining or oversampling techniques, achieved an accuracy of 92.2% (F1-score 0.837, AUC 0.962) on the unseen hold-out set. Cross-validation demonstrated stable baseline performance with an accuracy of 92.1 ± 3.0%, F1-score 0.810 ± 0.092, and AUC 0.946 ± 0.061. Supervised pretraining consistently improved performance relative to the baseline model across datasets. Table 2 confirms the aggregate benefit, with supervised pretraining raising pooled accuracy by 1.7%. Supervised pretraining on the Zhongshan-pretrain dataset yielded the greatest overall improvement, achieving 95.4% accuracy, F1-score 0.908 and AUC 0.981 on the test set. As shown in Table C in S1 Appendix, paired bootstrap resampling against the baseline model corresponded to statistically significant absolute improvements in performance (accuracy +3.2% [95% CI: 2.7, 3.8], F1-score +0.071 [95% CI: 0.059, 0.084], AUC + 0.019 [95% CI: 0.013, 0.025]) and calibration (Brier score -0.032 [95% CI: -0.037, -0.027]).

**Table 1:** Brugada classification model performance using the 25%-75% train-test split.

Pretraining method	Pretraining data	SMOTE	Acc. (%)	F1-score	AUC	Sensitivity	Specificity	NPV	PPV	Ave. prec.	Brier score
**Baseline**											
N	N	N	92.2	0.837	0.962	0.762	0.979	0.921	0.928	0.931	0.073
N	N	Y	92.1	0.840	0.964	0.789	0.968	0.928	0.898	0.930	0.073
**Supervised pretraining**											
Supervised	PTB-XL	N	92.4	0.841	0.971 *▲	0.770	0.979	0.923	0.929	0.938	0.067
Supervised	PTB-XL	Y	93.5	0.865	0.976 ^†^*▲*	0.793	0.985	0.931	0.950	0.950	0.060
Supervised	Zhongshan-pretrain	N	95.4 *▲	0.908	0.981 *▲	0.857	0.989	0.951	0.965	0.965	0.040
Supervised	Zhongshan-pretrain	Y	95.6 ^†^*▲*	0.913	0.981 ^†^*▲*	0.881	0.983	0.959	0.948	0.963	0.038
**Self-supervised pretraining**											
SimCLR	PTB-XL	N	92.7	0.855	0.957	0.822	0.964	0.939	0.890	0.908	0.063
SimCLR	PTB-XL	Y	91.8	0.835	0.959	0.791	0.963	0.928	0.885	0.917	0.069
SimCLR	Imperial	N	93.2 *▲	0.863	0.970 *▲	0.815	0.974	0.937	0.917	0.930	0.063
SimCLR	Imperial	Y	91.8	0.830	0.966	0.766	0.972	0.921	0.907	0.925	0.076
SimCLR	Zhongshan-pretrain	N	91.2	0.800	0.950	0.709	0.979	0.911	0.918	0.902	0.080
SimCLR	Zhongshan-pretrain	Y	91.1	0.816	0.940	0.756	0.966	0.917	0.887	0.863	0.077
MoCo-V2	PTB-XL	N	90.5	0.787	0.940	0.710	0.969	0.910	0.881	0.885	0.079
MoCo-V2	PTB-XL	Y	90.5	0.807	0.929 ^†^*▼*	0.752	0.960	0.916	0.869	0.859	0.081
MoCo-V2	Imperial	N	91.2	0.822	0.949	0.775	0.961	0.923	0.875	0.890	0.079
MoCo-V2	Imperial	Y	92.3	0.837	0.967 ^‡^*▲*	0.801	0.963	0.936	0.877	0.918	0.067
MoCo-V2	Zhongshan-pretrain	N	90.0 ^†^*▼*	0.790	0.955	0.713	0.967	0.905	0.885	0.892	0.081
MoCo-V2	Zhongshan-pretrain	Y	91.0 ^†^*▼*	0.811	0.963	0.739	0.970	0.913	0.898	0.911	0.076

Note: Y- Yes; N-No; SMOTE - synthetic minority oversampling technique; Acc. – Accuracy; AUC - Area under the receiver operating characteristic curve; NPV – Negative predictive value; PPV – Positive predictive value; Ave. prec. – Average precision.

* P < 0.002 comparing each model (no SMOTE) against the baseline (no SMOTE).

† *P < 0.002 comparing each model (SMOTE) against the baseline (SMOTE).*

‡ *P < 0.002 comparing each model (no SMOTE) with the same model (SMOTE).*

In the “AUC” column, significance was assessed with the Holm-corrected DeLong test. In the “Acc. (%)” column, significance was assessed with the Holm-corrected McNemar test. *▲ indicates improvement, ▼ indicates worsening.*

**Table 2:** Impact of pretraining approach and SMOTE oversampling on overall model performance.

Improvements	Pooled Accuracy*	Δ
Baseline model	92.2%	–
+ SMOTE	92.1%	-0.1
+ Supervised pretraining	93.9%	+1.7
+ Supervised pretraining with SMOTE	94.6%	+2.4
+ SimCLR	92.4%	+0.2
+ SimCLR with SMOTE	91.6%	-0.6
+ MoCo-V2	90.6%	-1.6
+ MoCo-V2 with SMOTE	91.3%	-0.9

*Pooled accuracy, including models pretraining using the Imperial, PTB-XL and Zhongshan-pretrain datasets. Δ, change in pooled accuracy.

**Table 3:** Brugada classification model false positive rate, evaluated on the external Brugada mimic dataset.

Pretraining method	Pretraining data	SMOTE	False Positives				
			RBBB (n = 200)	Early repolarisation (n = 200)	Anterior STEMI (n = 200)	HCM (n = 11)	Overall (n = 611)
**Baseline**							
N	N	N	58 (29%)	48 (24%)	48 (24%)	3 (27%)	157 (26%)
N	N	Y	71 (22%)	59 (16%)	52 (22%)	5 (45%)	187 (31%)
**Supervised pretraining**							
Supervised	PTB-XL	N	148 (74%)	100 (50%)	108 (54%)	6 (54%)	362 (59%)
Supervised	PTB-XL	Y	15 (8%)	8 (4%)	22 (11%)	0 (0%)	45 (7%)
Supervised	Zhongshan-pretrain	N	44 (22%)	32 (16%)	44 (22%)	2 (18%)	122 (20%)
Supervised	Zhongshan-pretrain	Y	131 (66%)	143 (72%)	137 (69%)	7 (63%)	418 (68%)
**Self-supervised pretraining**							
SimCLR	PTB-XL	N	15 (8%)	6 (3%)	8 (4%)	1 (9%)	30 (5%)
SimCLR	PTB-XL	Y	9 (5%)	8 (4%)	6 (3%)	0 (0%)	23 (4%)
SimCLR	Imperial	N	95 (48%)	118 (59%)	120 (60%)	6 (54%)	339 (56%)
SimCLR	Imperial	Y	102 (51%)	116 (58%)	127 (64%)	6 (54%)	351 (57%)
SimCLR	Zhongshan-pretrain	N	88 (44%)	69 (35%)	59 (30%)	4 (36%)	220 (36%)
SimCLR	Zhongshan-pretrain	Y	29 (15%)	21 (11%)	17 (9%)	0 (0%)	67 (11%)
MoCo-V2	PTB-XL	N	68 (34%)	39 (20%)	62 (31%)	2 (18%)	171 (28%)
MoCo-V2	PTB-XL	Y	79 (40%)	55 (28%)	80 (40%)	4 (36%)	218 (36%)
MoCo-V2	Imperial	N	96 (48%)	82 (41%)	90 (45%)	4 (36%)	272 (45%)
MoCo-V2	Imperial	Y	50 (25%)	42 (21%)	46 (23%)	2 (18%)	140 (23%)
MoCo-V2	Zhongshan-pretrain	N	4 (2%)	3 (2%)	1 (1%)	0 (0%)	8 (1%)
MoCo-V2	Zhongshan-pretrain	Y	11 (6%)	14 (7%)	8 (4%)	0 (0%)	33 (5%)

Note: Y- Yes; N-No; SMOTE - synthetic minority oversampling technique. Number of false positives by mimic category, false positive rate (%) in brackets. All models evaluated were trained using the 25%-75% train-test split.

Self-supervised pretraining performance varied more widely depending on the contrastive framework and pretraining dataset used, although most configurations performed similarly to the baseline model. The strongest self-supervised model was SimCLR pretrained on the Imperial dataset, which achieved 93.2% accuracy, AUC 0.970, and sensitivity 0.815 on the test set, representing a modest but statistically significant improvement over the baseline. SimCLR pretrained on the PTB-XL dataset yielded smaller gains in performance, although this did not reach significance by DeLong or McNemar testing. Paired bootstrapping comparison of SimCLR pretrained on PTB-XL against the baseline demonstrated a small but statistically significant improvement in F1-score and sensitivity, alongside a reduction in Brier score (**Table C in** S1 Appendix). MoCo-V2 models consistently underperformed SimCLR and, in several configurations, showed slight degradation relative to baseline, likely reflecting greater sensitivity to optimisation and calibration. Cross-validation results were concordant with test set findings, with supervised pretraining on the Zhongshan-pretrain dataset outperforming all alternatives across performance metrics and self-supervised models using the SimCLR framework demonstrating smaller and less consistent improvements.

The application of SMOTE increased the minority class (Brugada ECGs) from 594 to 1,772 individual beats, however its impact on model performance was inconsistent. Some configurations showed small gains and others demonstrated no improvement or degraded performance. Cross-validation results largely showed overlapping confidence intervals, indicating no reliable or generalisable benefit.

When the models were re-trained using a conventional train-test split of 80%-20%, the benefits of pretraining were less pronounced on the hold-out test set, as shown in **Table D in** S1 Appendix. The baseline model achieved an accuracy of 96.6% and an AUC score that was close to 1.0, leaving little room for pretrained models to demonstrate significant improvements. Learning curve analysis shown in **Fig A in** S1 Appendix further illustrates this trend, demonstrating that the relative benefit of supervised pretraining diminishes as the number of labelled training samples increases and baseline performance approaches a ceiling. The cross-validation baseline model performance for the 80%-20% split (**Table E** in S1 Appendix) was more consistent, with narrower confidence intervals than for the 25%-75% split. Application of SMOTE using the 80%-20% train-test split yielded an additional 3,664 synthetic Brugada ECGs to the training set (from 1,989–5,653). However, as with the 25%-75% train-test split, there was no consistent improvement in performance when using SMOTE-generated ECGs.

When evaluated against an external “Brugada mimic” test set (Table 3), the baseline model yielded an overall false positive rate (FPR) of 26%. Specific self-supervised pretrained model configurations dramatically improved the FPR to 5% and below, specifically the SimCLR-pretrained models using both the PTB-XL and dataset and the MoCo-V2-pretrained models using the Zhongshan-pretrain dataset. In contrast, supervised pretrained models exhibited variable performance, with some configurations exhibiting a similar FPR to the baseline and others showing a substantially higher FPR Table 3.

**Fig 6:**
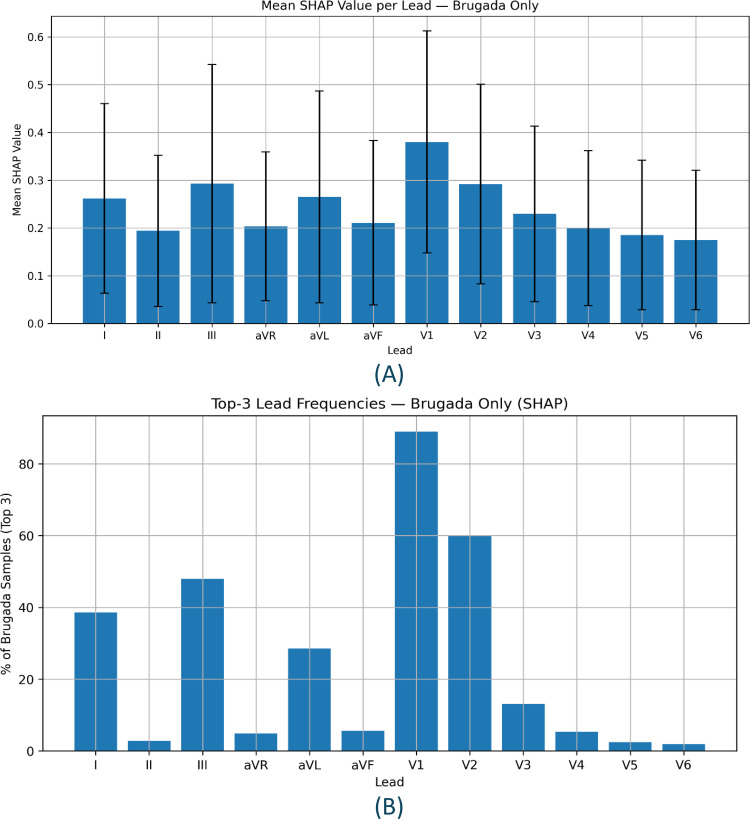
SHAP-based lead-level analysis for Brugada syndrome classification. (A) Mean absolute SHAP value per ECG lead across all test samples with Brugada syndrome, reflecting average attribution magnitude. Error bars represent one standard deviation. (B) Frequency with which each lead appeared in the top three most impactful, as determined by SHAP attribution, across Brugada cases.

By applying SHAP analysis to the Brugada ECG samples in the test set, the relative contribution of each lead to the model’s predictions was determined for the positive class, i.e., whether a Brugada ECG is present. SHAP values aggregated across each lead showed the highest mean values in leads V1 and V2 (Fig 6A). The frequency with which a given lead was ranked within the top 3 leads by summed SHAP values was also determined (Fig 6B), demonstrating again that leads V1 (89% of cases) and V2 (60% of cases) were the two most influential leads for the model’s decision making for identifying Brugada ECGs. This mirrors clinical practice, whereby leads V1 and V2 situated over the right ventricular outflow tract, are the essential leads for determining the presence of a Brugada ECG pattern.

To validate that these lead-level attributions reflected meaningful learned representations, we performed additional explainability sanity checks. In **Fig B in** S1 Appendix, progressive parameter randomisation degraded both model performance and SHAP attribution similarity toward chance, indicating that explanations were model-dependent rather than artefactual, while lead ablation showed the largest changes in predicted Brugada ECG probability when masking V1-V2. In **Fig C in** S1 Appendix, time-resolved SHAP overlays show that correctly classified Brugada ECGs exhibit concentrated attribution in leads V1–V2, primarily around the QRS complex, ST segment, and early T wave, whereas misclassified cases show attribution spread across other leads, such as III and aVL. This indicates that correct predictions rely on clinically expected Brugada features, while misclassifications arise when the model attends to less informative or atypical ECG regions.

## Discussion

In this study, we evaluated the utility of various pretraining approaches and synthetic data generation on the performance of an AI-ECG classification model for Brugada syndrome. We intentionally restricted the amount of training data available by partitioning a much larger proportion of our available data into the test set, simulating the data scarcity encountered when developing models for rare diseases. We found that the supervised pretrained approach consistently outperformed the baseline model performance in the context of severely restricted training data. Additionally, some self-supervised approaches also achieved competitive performance beyond the baseline model, but these gains were highly dependent on the framework and pretraining dataset used.

### Baseline model performance

The baseline model, which incorporated neither pretraining nor oversampling, achieved an accuracy of 92.2% and an F1-score of 0.837 on the test set, despite its training being intentionally restricted to a relatively small pool of data (ECG samples from only 44 positive Brugada cases). Although this seems a reasonably high level of accuracy, this was a partition of a single dataset, not an external testing set. Despite the high accuracy of the baseline model, we were able to demonstrate considerable improvements in model performance, mitigating for limited training data, using several machine learning strategies.

### Supervised pretraining

Both supervised pretraining approaches (PTB-XL and Zhongshan-pretrain) significantly outperformed the baseline non-pretrained approach, showing strong improvements in accuracy, specificity, and AUC across both test and cross-validation metrics. With the addition of supervised pretraining, the overall accuracy of the model increased by up to 3.2% (from 92.2% to 95.4%) using the Zhongshan-pretrain dataset, with increased sensitivity (from 0.762 to 0.857) while maintaining high specificity (from 0.979 to 0.989). If this improvement was borne out in a prospective, external validation study, this would result in a substantial, clinically meaningful, reduction in misdiagnoses by the final model. Taking the example of 1000 patients referred for suspected Brugada syndrome and assuming a 25% prevalence of Brugada syndrome within this high-risk cohort, the supervised pretrained model would detect 24 additional true Brugada cases that may have otherwise been missed as false negatives (9.5% improved sensitivity). This would roughly half the number of patients who received false reassurance and ensure that these true Brugada cases are appropriately flagged for timely ICD implantation or lifestyle interventions, mitigating their risk of sudden death. An important caveat is that the positive predictive value of the developed models would be adversely affected in a lower prevalence context, such as screening unselected ECGs from the electronic health record or emergency department presentations. For this reason, these models should be deployed in contexts where there is a high pre-test probability, such as patients referred to inherited cardiac condition specialist clinics or undergoing evaluation for suspected Brugada syndrome with ambulatory 24-hour 12-lead Holter monitoring.

The PTB-XL and Zhongshan-pretrain datasets both comprise thousands of labelled ECGs displaying Brugada mimics, including RBBB and ST elevation myocardial infarction. True Brugada ECG patterns can be easily mistaken for these Brugada mimics even by human experts [18–21]. Pretraining using the Zhongshan-pretrain dataset achieved higher accuracy versus the PTB-XL dataset, which likely reflects its closer similarity to the target domain data and the focused inclusion of RBBB cases, the key differential diagnosis for Brugada syndrome. Our findings suggest that supervised pretraining using ECGs resembling positive cases helps neural networks learn generalisable features that improve performance during fine-tuning.

### Self-supervised pretraining

Self-supervised pretraining approaches performed variably on the unseen test set, but specific configurations did improve performance beyond the baseline model, although not achieving the same performance as the best supervised model. The variability in performance improvement likely relates to the inherent nature of self-supervised pretraining, where the features learnt by the model during pretraining are not optimised towards the downstream task of Brugada ECG classification, in contrast with supervised pretraining. Instead, the models learn more generic underlying patterns and features from the ECG signal, which can vary.

Despite this variation and the disadvantage of unlabelled ECG data, the best performing self-supervised model (SimCLR framework applied to the Imperial dataset), achieved an accuracy of 93.2% – comparable to the supervised pretrained models (92.4%-95.4%). This result is particularly impactful, as it suggests that effective model pretraining can be readily achieved using entirely unlabelled datasets, bypassing the need for manual, resource-intensive labelling processes [22].

The choice of pretraining dataset and approach appeared to strongly influence the extent to which pretraining improved accuracy. For example, the Imperial dataset showed more reliable performance on the test set as compared to the Zhongshan-pretrain and PTB-XL datasets, likely due to the Brugada-specific ECG data contained within it. Conversely, self-supervised models pretrained using the Imperial dataset generally had a disproportionately high FPR on the external Brugada mimic stress test, in contrast to models pretrained using the larger and more diverse PTB-XL and Zhongshan-pretrain datasets, where some configurations yielding substantial FPR improvements over the baseline model. This suggests that the lack of labels in self-supervised pretraining renders model performance more dependent on the diversity, representativeness and quality of the pretraining dataset [23]. As with supervised pretraining and oversampling, the effectiveness of the self-supervised pretraining frameworks likely depends on how well the data augmentations capture meaningful variations relevant to Brugada classification; however, where diagnostically relevant morphological features are obscured, performance of these self-supervised pretrained models may be degraded [24]. Collectively, these findings indicate that self-supervised pretraining is not inherently advantageous but can potentially make models more robust to distribution shifts and help mitigate false positives. However, this is contingent on careful selection of pretraining data and framework.

The benefits of pretraining were profoundly reduced in the models trained using the 80%-20% train test split, and although some models showed numerically higher accuracy or AUC, these differences were marginal and do not translate into meaningful gains over the baseline model. **Tables F in** S1 Appendix and **Table G in** S1 Appendix summarise the computational cost of supervised and self-supervised pretraining across datasets of different sizes, demonstrating that training cost scales strongly with the size of the dataset. Given the additional computational overhead of pretraining, marginal performance gains and excellent baseline model performance, these results do not support the use of pretraining when adequate labelled data is available.

### Oversampling

Under data-scarce conditions, the addition of SMOTE-generated ECGs had a modest and inconsistent effect on overall model performance across the different approaches. In the baseline models, its use resulted in almost no meaningful change, with accuracy and F1-score remaining essentially stable. Among both supervised and self-supervised pretrained models, the results were variable, with only the MoCo-V2 model pretrained on the Imperial dataset accruing an improved overall performance.

Synthetic Brugada ECGs were generated using SMOTE to augment the training set and to address class imbalance within the training data. Addressing class imbalance is especially pertinent in real-life ECG datasets, with normal conditions like sinus rhythm ECGs, and common mild abnormalities like RBBB, vastly over-represented compared to Brugada syndrome and other rare conditions [25]. By addressing the class imbalance, SMOTE can potentially help AI-ECG models better represent the minority classes but also reduce the risk of bias, ensuring more accurate predictions across all classes.

However, SMOTE has limitations in terms of diversity. By interpolating existing minority class samples, it tends to generate synthetic data that closely resembles the real samples, without introducing novel variations. To assess this, we conducted a t-SNE analysis on Brugada ECGs, comparing real and synthetic samples, shown in **Fig D in** S1 Appendix. This demonstrated that SMOTE ECGs clustered with real patient ECGs, indicating morphological plausibility but also a lack of diversity, with the possibility of overfitting. While SMOTE addresses class imbalance, its conservative nature may limit the introduction of more diverse examples that could enhance the model’s robustness [20].

SMOTE was chosen over other data augmentation techniques because it provides a straightforward, effective way to address class imbalance and introduce synthetic variability. While waveform-specific augmentations, such as noise, temporal warping or lead masking, could potentially enhance diversity, they require careful selection to ensure generated data remains realistic and avoid altering diagnostically important features which could lead to ECG misclassification. This is particularly important in the case of Brugada syndrome, which is characterised by the specific morphological appearance of coved or saddleback ST elevation in the V1-V3 leads, where distortion of the signal may inadvertently alter the ECG diagnosis.

In addition to SMOTE, we also trialled a convolutional variational autoencoder (VAE) with skip connections and standard reconstruction and KL divergence loss to generate synthetic Brugada ECGs (see Section “Synthetic **ECG** generation using a variational autoencoder” in S1 Appendix). While the model reproduced some lead-wise morphology (**Fig E in** S1 Appendix), the generated signals lacked coherent inter-lead relationships and were ultimately deemed unsuitable for training after expert review. However, this does not imply that all deep generative models are unsuitable for generating synthetic ECGs. For example, Zanchi et al demonstrated that GAN-generated Brugada ECGs could be indistinguishable from real cases based on expert evaluation, with low mean accuracy (40%) and wide inter-rater variability in distinguishing synthetic from real ECGs [26]. These results suggest that deep generative models can produce high-quality synthetic ECGs to handle class imbalance or data scarcity, but further work is still necessary to refine these models, possibly by incorporating domain-specific constraints and regularisation strategies to ensure physiological plausibility and preserve the inter-lead relationships critical to accurate ECG interpretation [27]. Future work within this field should focus on the validation of synthetic ECG data generated using a range of augmentation, generative and oversampling techniques.

### AI-ECG for Brugada syndrome

Several groups have developed machine learning approaches to automate the detection of the Brugada ECG pattern from the 12-lead ECG. Liu et al first explored the utility of supervised pretraining to discriminate RBBB ECGs from non-RBBB ECGs, and subsequently applied transfer learning to a Brugada classification model [9]. Their training dataset comprised 138 ECGs with a type 1 Brugada ECG pattern and 138 non-Brugada ECGs. The diagnostic performance of the model was compared with that of board-certified cardiologists, achieving an AUC of 0.96, with 88.4% sensitivity and 89.1% specificity, showing high consistency with standard diagnoses (kappa coefficient 0.78). Liao et al leveraged a much larger dataset consisting of 1190 12-lead ECGs of patients with definite and suspected Brugada syndrome [28]. Their Brugada type 1 classification model achieved impressive performance on the test cohort, achieving an AUC of 0.976, comparable to two independent cardiologists’ interpretation. The developed Brugada AI-ECG classification models were also applied to 24-hour 12-lead Holter recordings, highlighting a potential use case of these models for ambulatory ECG diagnostics [28]. Ronan et al evaluated a self-supervised pretraining approach for Brugada ECG classification using the VICReg framework [29]. They utilised almost four million unlabelled ECGs for model pretraining and used a training set of 142 Brugada ECGs and 258 non-Brugada ECGs, reporting a superior performance of the VICReg fine-tuned model compared to the baseline model (AUC 0.88 vs 0.76). In this study, we leveraged a much broader range of techniques, directly comparing both supervised and self-supervised approaches, as well as oversampling techniques not previously applied for Brugada classification, all within the context of intentionally restricted data.

### Rare disease implications

Rare conditions, though individually uncommon, collectively represent a substantial public health burden. Patients with rare conditions are subject to health inequalities due to delays to diagnosis, limited treatment options and under-awareness of their condition [30]. As AI-ECG models are developed, it is essential to prioritise equitable deployment to prevent further widening of health disparities for patients with rare diseases.

Large pretrained foundational models can facilitate the development of AI-ECG models for detecting other rare diseases with small datasets. For example, Hu et al leveraged supervised pretraining and transfer learning to improve AI-ECG model performance to detect conditions like carcinoid syndrome and pericardial constriction, even though these conditions were not part of the initial training data [31]. Rare diseases like Brugada syndrome present a particular equity challenge, as their low prevalence makes it difficult to curate sufficiently large and diverse labelled datasets. This increases the risk of overfitting to the limited cases available, which may not capture the full range of ECG variation of the condition. Preliminary supervised or self-supervised pretraining on large, heterogeneous datasets that include diverse populations and related ECG phenotypes can help address this gap, improving generalisability and equity once models are fine-tuned on smaller, disease-specific datasets. Validation of rare disease AI-ECG models on sufficiently diverse datasets is an important next step to ensure that they perform accurately and reliably across different demographic groups.

The clinical setting in which AI-ECG models are deployed is also highly relevant when evaluating their real-world performance. The composition of our training and test set had a much higher proportion of positive Brugada cases (1:3) relative to what would be expected in the general population. As a result, testing the model in a real-world setting with lower Brugada syndrome prevalence may reduce its precision (positive predictive value). However, a higher prevalence of true Brugada cases could be encountered in different clinical contexts, such as in patients with a positive family history, suspicious presentation or suspected cases undergoing 12-lead ambulatory ECG monitoring. In these cases, the class distribution of our training set would be more representative of the real-world class distribution.

### Limitations

The training and test sets were derived from a single centre and comprised only normal, right bundle branch block and Brugada ECGs. While this design allowed controlled evaluation under data-scarce conditions, it does not fully reflect the full diversity of ECG morphologies encountered in clinical practice. To partially address this, we evaluated model behaviour using a small external Brugada mimic stress test sourced from a different centre. Although this provided useful insight into out-of-domain false positive behaviour, the limited size of the mimic dataset restricts the precision of performance estimates and precludes robust subgroup analyses. As such, conclusions regarding generalisability to broader Brugada-like pathologies should be interpreted cautiously, and validation against larger independent external datasets remains necessary.

As discussed, the overrepresentation of Brugada cases in the Zhongshan-baseline dataset does not reflect the true distribution within the general population. Applying these models to our Brugada enriched test set is therefore likely to have inflated their performance. The developed models have only undergone internal validation testing and thus there is likely to be a degree of overfitting of the data. Several sources of heterogeneity and bias were not fully captured as part of this study, including variability of ECG machines from different vendors, different ECG amplifier/filter settings, ECG electrode placement differences (including conventional vs high praecordial lead ECG placement), label noise due to retrospective data curation, and potential demographic drift.

We do not presume that the performance of the models trained in this study reflect performance in a real-world setting against prospective cases. Rather, these models serve to demonstrate how different machine learning techniques can mitigate against data scarcity for ECG classification tasks. Our findings suggest that implementation of these adjunctive machine learning techniques has a positive impact on model performance, enabling AI-ECG models to better overcome the challenge of data scarcity during training.

SMOTE addressed class imbalance effectively, but it did so by generating synthetic samples that closely resembled real data without introducing significant diversity. This could limit the model’s ability to generalise and lead to overfitting. SMOTE-generated ECGs were reviewed for biological plausibility by only a single expert. A more robust and systematic approach to generation and validation of synthetic ECG data may ensure consistency and maintain data integrity.

## Conclusion

Our findings demonstrate that by leveraging appropriate pretraining approaches and oversampling techniques, it is possible to significantly improve the performance of AI-ECG models for detecting Brugada syndrome, even with limited training data. This is a viable strategy for developing AI-ECG models for other rare cardiac conditions where data is scarce, and these approaches should be considered during model development to maximise accuracy and performance prior to clinical deployment.

## Supporting information

S1 Appendix Supplementary methods and results. (DOCX)

## References

[1] Attia ZI, Harmon DM, Behr ER, Friedman PA. Application of artificial intelligence to the electrocardiogram. Eur Heart J. 2021;42 (46):4717–30. doi: 10.1093/eurheartj/ehab649 34534279 PMC8500024

[2] Li C, Denison T, Zhu T. A Survey of Few-Shot Learning for Biomedical Time Series. IEEE Rev Biomed Eng. 2025;18:192–210. doi: 10.1109/RBME.2024.3492381 39504299

[3] Pezoulas VC, Zaridis DI, Mylona E, Androutsos C, Apostolidis K, Tachos NS, et al. Synthetic data generation methods in healthcare: A review on open-source tools and methods. Comput Struct Biotechnol J. 2024;23:2892–910. doi: 10.1016/j.csbj.2024.07.005 39108677 PMC11301073

[4] Postema PG. About Brugada syndrome and its prevalence. Europace. 2012;14 (7):925–8. doi: 10.1093/europace/eus042 22417721

[5] Asatryan B, Barth AS. Sex-related differences in incidence, phenotype and risk of sudden cardiac death in inherited arrhythmia syndromes. Front Cardiovasc Med. 2023;9:1010748. doi: 10.3389/fcvm.2022.1010748 36684594 PMC9845907

[6] Milman A, Andorin A, Postema PG, Gourraud J-B, Sacher F, Mabo P, et al. Ethnic differences in patients with Brugada syndrome and arrhythmic events: New insights from Survey on Arrhythmic Events in Brugada Syndrome. Heart Rhythm. 2019;16 (10):1468–74. doi: 10.1016/j.hrthm.2019.07.003 31284050

[7] Wagner P, Strodthoff N, Bousseljot R-D, Kreiseler D, Lunze FI, Samek W, et al. PTB-XL, a large publicly available electrocardiography dataset. Sci Data. 2020;7 (1):154. doi: 10.1038/s41597-020-0495-6 32451379 PMC7248071

[8] Silva I, Moody GB. An Open-source Toolbox for Analysing and Processing PhysioNet Databases in MATLAB and Octave. J Open Res Softw. 2014;2 (1):e27. doi: 10.5334/jors.bi 26525081 PMC4627662

[9] Liu CM, Liu CL, Hu KW, Tseng VS, Chang SL, Lin YJ, et al. A Deep Learning–Enabled Electrocardiogram Model for the Identification of a Rare Inherited Arrhythmia: Brugada Syndrome. *Canadian Journal of Cardiology*. 2022;38 (2): 152–9. 10.1016/J.CJCA.2021.08.014 34461230

[10] Chen T, Kornblith S, Norouzi M, Hinton G. A simple framework for contrastive learning of visual representations. In: 2020. 1575–85. https://arxiv.org/pdf/2002.05709

[11] Chen X, Fan H, Girshick R, He K. Improved baselines with momentum contrastive learning. 2020. https://arxiv.org/pdf/2003.04297

[12] Liu H, Zhao Z, She Q. Self-supervised ECG pre-training. Biomedical Signal Processing and Control. 2021;70:103010. doi: 10.1016/j.bspc.2021.103010

[13] Abubaker MB, Babayiğit B. Detection of Cardiovascular Diseases in ECG Images Using Machine Learning and Deep Learning Methods. IEEE Trans Artif Intell. 2023;4 (2):373–82. doi: 10.1109/tai.2022.3159505

[14] Mehari T, Strodthoff N. Self-supervised representation learning from 12-lead ECG data. Comput Biol Med. 2022;141:105114. doi: 10.1016/j.compbiomed.2021.105114 34973584

[15] Pandey SK, Janghel RR. Automatic detection of arrhythmia from imbalanced ECG database using CNN model with SMOTE. Australas Phys Eng Sci Med. 2019;42 (4):1129–39. doi: 10.1007/s13246-019-00815-9 31728941

[16] Kim YK, Lee M, Song HS, Lee S-W. Automatic Cardiac Arrhythmia Classification Using Residual Network Combined With Long Short-Term Memory. IEEE Trans Instrum Meas. 2022;71:1–17. doi: 10.1109/tim.2022.3181276

[17] Adebayo J, Gilmer J, Muelly M, Goodfellow I, Hardt M, Kim B. In: Proceedings of the 32nd International Conference on Neural Information Processing Systems, 2018. 9525–36.

[18] Chhabra L, Spodick DH. Brugada pattern masquerading as ST-segment elevation myocardial infarction in flecainide toxicity. Indian Heart J. 2012;64 (4):404–7. doi: 10.1016/j.ihj.2012.06.010 22929826 PMC3861284

[19] Surawicz B. Brugada syndrome: manifest, concealed, “asymptomatic,” suspected and simulated. J Am Coll Cardiol. 2001;38 (3):775–7. doi: 10.1016/s0735-1097(01)01472-3 11527632

[20] Khan AR, Waqar S, Arif A, Ul Haq F, Shah MI. Brugada Syndrome Misdiagnosed As Acute Myocardial Infarction: A Case Report. Cureus. 2022;14 (7):e26998. doi: 10.7759/cureus.26998 35989735 PMC9386327

[21] Gottschalk BH, Anselm DD, Brugada J, Brugada P, Wilde AA, Chiale PA, et al. Expert cardiologists cannot distinguish between Brugada phenocopy and Brugada syndrome electrocardiogram patterns. Europace. 2016;18 (7):1095–100. doi: 10.1093/europace/euv278 26498159

[22] Spathis D, Perez-Pozuelo I, Marques-Fernandez L, Mascolo C. Breaking away from labels: The promise of self-supervised machine learning in intelligent health. Patterns (N Y). 2022;3 (2):100410. doi: 10.1016/j.patter.2021.100410 35199063 PMC8848012

[23] Hammoud HAAK, Das T, Pizzati F, Torr P, Bibi A, Ghanem B. On Pretraining Data Diversity for Self-Supervised Learning. 2024. doi: 10.1007/978-3-031-72992-8_4

[24] Morningstar W, Bijamov A, Duvarney C, Friedman L, Kalibhat N, Liu L, et al. Augmentations vs Algorithms: What Works in Self-Supervised Learning. 2024; http://arxiv.org/abs/2403.05726

[25] Liu Y, Li Q, Wang K, Liu J, He R, Yuan Y, et al. Automatic Multi-Label ECG Classification with Category Imbalance and Cost-Sensitive Thresholding. Biosensors (Basel). 2021;11 (11):453. doi: 10.3390/bios11110453 34821669 PMC8615597

[26] Zanchi B, Monachino G, Faraci FD, Metaldi M, Brugada P, Sarquella-Brugada G, et al. Synthetic electrocardiograms for Brugada syndrome: from data generation to expert cardiologists evaluation. Eur Heart J Digit Health. 2025;6 (4):683–7. doi: 10.1093/ehjdh/ztaf039 40703115 PMC12282356

[27] Zanchi B, Monachino G, Fiorillo L, Conte G, Auricchio A, Tzovara A, et al. Synthetic ECG signals generation: A scoping review. Comput Biol Med. 2025;184:109453. doi: 10.1016/j.compbiomed.2024.109453 39612827

[28] Liao S, Bokhari M, Chakraborty P, Suszko A, Jones G, Spears D, et al. Use of Wearable Technology and Deep Learning to Improve the Diagnosis of Brugada Syndrome. JACC: Clinical Electrophysiology. 2022;8 (8): 1010–20. 10.1016/J.JACEP.2022.05.003 35981788

[29] Ronan R, Tarabanis C, Chinitz L, Jankelson L, Charney LH. Brugada ECG detection with self-supervised VICReg pre-training: a novel deep learning approach for rare cardiac diseases. medRxiv. 2024; 2024.03.29.24305072. 10.1101/2024.03.29.24305072

[30] Kole A, Faurisson F. Rare diseases social epidemiology: analysis of inequalities. Adv Exp Med Biol. 2010;686:223–50. doi: 10.1007/978-90-481-9485-8_14 20824449

[31] Hu SM, Barrios JP, Tison GH. A deep foundation model for electrocardiogram interpretation: enabling rare disease detection through transfer learning. Eur Heart J Digit Health. 2025;6 (4):619–23. doi: 10.1093/ehjdh/ztaf051 40703125 PMC12282392

