# Supplementary material for: AI-ECG classification for Brugada syndrome: A study of machine learning techniques to optimise for limited datasets
Source: PLOS Digit Health. 2026 Feb 25;5(2):e0001222. doi: 10.1371/journal.pdig.0001222 (PMC12935214; doi:10.1371/journal.pdig.0001222)
Supplement: S1 Appendix — (DOCX) [file pdig.0001222.s001.docx]

Supplementary Appendix

# Table of contents

[Supplementary Methods 3](#_Toc206157000)

[ECG Processing 3](#_Toc206157001)

[Baseline Model Architecture 4](#_Toc206157002)

[Supervised pretraining 6](#_Toc206157003)

[Data augmentation 6](#_Toc206157004)

[Augmentation ablation study using SimCLR 10](#_Toc206157004)

SMOTE [Oversampling 11](#_Toc206157005)

[Supplementary Results 12](#_Toc206157006)

Table A 12

Table B 13

Table C 15

Table D 16

Table E 17

Table F 19

Table G 20

Fig A 21

Fig B 22

Fig C 23

Fig D 24

Synthetic ECG generation using a variational autoencoder 25

Fig E 26

## **Supplementary Methods**

All codes are available at <https://github.com/raaifhadadi/brugada-ecg-representation-learning>

### ECG Processing

To prepare ECG data for analysis and model training, several steps were required as part of the data processing pipeline:

1. **Downsampling**

The sampling rate for all ECG recordings was uniformly downsampled to 100Hz. The Zhongshan dataset and Vanderbilt University Medical Center ECGs were originally acquired at 500Hz and were downsampled by first applying a 5-point moving-average (boxcar) low-pass filter to suppress high-frequency components and prevent aliasing, followed by decimation by a factor of five. BARD ECGs were originally sampled at 1,000 Hz and were similarly downsampled to 100Hz using a 10-point moving-average low-pass filter and decimation by a factor of ten. The PTB-XL dataset provides waveforms at both 500Hz and 100Hz; for consistency with the preprocessing pipeline, the officially released 100Hz recordings were used directly.

Although this represents a substantial reduction in sampling rate for some datasets, the ECG features relevant to Brugada syndrome such as the QRS complex, J-point, and early ST-segment morphology, occur on the scale of tens to hundreds of milliseconds and are therefore preserved at 100Hz. This choice represents a deliberate trade-off to reduce data dimensionality and computational burden while retaining clinically meaningful information.

1. **Normalisation**

ECG signal amplitude values were normalised using z-score normalisation, applied separately to each lead. The mean and standard deviation for each lead were computed across all samples and time points within the training or test set.

1. **QRS Detection and Segmentation**

R-peaks were detected using the XQRS algorithm from the WFDB package, applied independently to each lead at 100Hz. XQRS identifies candidate beats using a combination of signal transformations, including squaring and moving-window integration.
Candidate R-peak times from all leads were merged using a multi-lead consensus strategy. Detected peaks were grouped if they occurred within ± 0.2 s (± 20 samples) of each other across leads. Groups with detections from fewer than three leads were discarded to reduce spurious peak detections and enforce physiological consistency. For each remaining group, the final consensus R-peak time was defined as the mean of the contributing lead-specific peak times.

From each consensus R-peak, a fixed 1.0-second window (0.5s before and 0.5s after the R-peak) was extracted across all twelve leads, yielding a 100 × 12 matrix per beat. This window length reliably captures the full QRS complex, the J-point, and early ST-segment and T-wave morphology, including the characteristic coved or saddleback ST-segment elevation patterns associated with Brugada syndrome, while minimising overlap between adjacent beats.

Segmenting ECGs into individual beats reduces the influence of beat-to-beat variability and allows the classification model to focus on intrinsic single-beat morphological features, providing uniform input length and structure for model training.

Baseline Model Architecture
A 1D convolutional neural network with DenseNet-style blocks was implemented for the binary classification task, as illustrated in Figure 1. While alternative architectures such as temporal convolutional networks and lightweight transformers may offer advantages in larger-scale settings, our pilot experiments suggested that, under severe data scarcity, the CNN backbone yielded more reliable discrimination–calibration trade-offs. Accordingly, we focused subsequent analyses on this architecture.

The model takes in downsampled ECG input. It begins with an initial convolutional block, followed by ten DenseNet blocks using concatenated skip connections. The number of filters was kept constant across all blocks to limit model complexity and reduce the risk of overfitting, given that the dataset is quite small (25%-75% split has 413 ECG beats) and one-dimensional. Global average pooling was applied to aggregate temporal features, followed by a fully connected layer with Leaky ReLU activation and dropout (rate 0.1), defining the feature extraction stage. A final dense layer with sigmoid activation produced the probability of Brugada versus non-Brugada ECG. The resulting network comprised 111,697 parameters and required approximately 4.2 x 10^6^ floating-point operations (FLOPs) per beat at inference.

The model was trained using the Adam optimiser (learning rate 1 × 10⁻⁴; β₁ = 0.9, β₂ = 0.999; no weight decay) with binary cross-entropy loss. No label smoothing was applied. Class weighting was evaluated as part of preliminary experiments but did not improve performance relative to oversampling strategies and was therefore not used in the final models unless explicitly stated. A batch size of 64 was used throughout. Early stopping was employed based on validation loss, with a minimum of 10 training epochs, a patience of 15 epochs, restoration of the best-performing weights and the maximum number of epochs was set to 100.

To assess generalisation performance, 8-fold cross-validation was performed using patient-level splits to prevent data leakage. Following cross-validation, the model was retrained on the full training set using the same configuration and evaluated once on the independent hold-out test set. A fixed decision threshold of 0.5 was used for classification.

### Supervised pretraining

To address the challenge of limited labelled data, we trained two separate one-dimensional convolutional neural networks with DenseNet-style blocks, each using a different large, labelled ECG dataset. The first model was trained on the Zhongshan-pretrain dataset to classify ECGs as either normal or showing RBBB. The second model was trained on the PTB-XL dataset to perform five-class classification of common ECG abnormalities.

Since PTB-XL follows a multilabel structure, where each ECG recording can be annotated with multiple diagnostic labels, we treated this as a multilabel classification task. The model used a sigmoid-activated dense output layer with five units, allowing it to assign independent probabilities to each diagnostic class. Binary cross-entropy was used as the loss function, which is well-suited for multilabel settings where labels are not mutually exclusive.

Both models used the same feature extractor architecture as our downstream Brugada classifier, shown in Figure 1, which illustrates the architecture used for binary classification. Pretraining was conducted until the loss plateaued, using the Adam optimiser with a learning rate of 0.0001 and a batch size of 64. After pretraining, the feature extractor weights from each model were transferred to a new model for Brugada detection. During fine-tuning, only the classification head and the final two DenseNet blocks were unfrozen. This is illustrated in Figure 2.

### Data augmentation

During self-supervised contrastive learning, positive pairs were created by applying independent augmentations to each ECG segment. These paired views guided the contrastive loss, encouraging the model to learn robust and invariant representations. To maintain physiological plausibility, each view was perturbed using a combination of baseline drift and additive Gaussian noise.

Baseline drift $d\left( t \right)$ was introduced by adding a sinusoidal waveform to each lead. This waveform is intended to approximate low-frequency fluctuations often observed in ECGs, such as those caused by respiration or electrode instability. The augmented signal $\tilde{x}_{t, l}$ for time $t$ and lead $l$ was defined as:

$$\tilde{x}_{t, l}=x_{t,l}+k\cdot IQR_{l} \cdot d(t)$$

$$d\left( t \right)=sin(2\pi\frac{t+\phi}{\lambda})$$

where λ is the wavelength of the sinusoid, randomly sampled from 300 to 500 samples (equivalent to 3–5 seconds at 100 Hz), and *ϕ* is a random phase offset sampled uniformly from the range [0, λ]. The sinusoid was scaled by the interquartile range (IQR) of each lead’s amplitude to preserve lead-specific variability and further multiplied by a global strength factor, $k$, sampled from a range between 1.5 to 2.5. This formulation ensures that the drift remains physiologically plausible while introducing realistic variability across the 12-lead ECG.

To simulate high-frequency noise commonly introduced by environmental or hardware sources, white Gaussian noise was added independently to each lead, given as:

$$\tilde{x}_{t, l}=x_{t,l}+ \epsilon_{t,l}$$

where $\epsilon_{t,l}$ ~ 𝒩(0, $\sigma_{l}^{2}$), and the standard deviation, $\sigma_{l}$ was calculated using:

$$\sigma_{l}=m\cdot STD_{lead}$$

where $m$is a scaling factor sampled from the range 0 to 0.2 and ${STD}_{lead}$ is the lead-specific standard deviation.

In addition, with 50% probability, one of the 12 ECG leads was randomly selected and zeroed out entirely. This channel dropout simulates lead disconnection or signal dropout, improving the model’s robustness to partial input failure. Global amplitude variability was also introduced through gain scaling, in which all leads of an ECG beat were multiplied by a random scalar sampled uniformly between 0.7 and 1.3 using:

$$\tilde{x}_{t, l}=g\cdot x_{t,l}$$

To simulate rare but clinically realistic polarity errors arising from lead misplacement, a single ECG lead was randomly selected and inverted by multiplying its signal by -1, given as:

$$\tilde{x}_{t, l}=- x_{t,l}$$

All other leads were left unchanged. Mild global temporal distortions were introduced using a time-warping augmentation that uniformly stretched or compressed the ECG beat around its centre, given as:

$$t'=c+(t-c)\cdot w$$

$$c=\frac{T-1}{2}, w=1+\delta$$

where $t'$ denotes warped time index, t denotes the original time index, $T$ denotes the number of samples, $c$ is the temporal centre of the signal, $w$ is the warp factor sampled between a continuous uniform distribution over the interval [-0.2, 0.2]. Signals were resampled using interpolation to preserve the original signal length.

Small rigid temporal shifts were applied to simulate minor misalignments in beat segmentation. A shift Δt was sampled uniformly from the range [-5, 5] samples, and the signal was shifted accordingly, with edge padding applied to maintain constant length. This preserves waveform shape while introducing small alignment variability.

Narrow-band periodic interference was simulated by adding a sinusoidal waveform at either 50 or 60Hz to each lead. The augmented signal was defined as:

$$\tilde{x}_{t, l}=- x_{t,l}+a_{l}sin(2\pi ft)$$

$$a_{l}=0.1 . STD_{l}$$

where f ∈ {50. 60} Hz and the amplitude and the amplitude $a_{l}$ is proportional to the standard deviation of lead $l$. This augmentation simulates power-line noise observed in clinical and ambulatory ECG recordings and introduces structured periodic interference distinct from uncorrelated noise.

SimCLR models employed the same model architecture proposed in Figure 1 (main manuscript), but with the classifier head replaced by a projection head comprising two dense layers with ReLU activation, respectively. Projection dimensions of 64 and 32 were used. Training was conducted using the Normalised Temperature-Scaled Cross-Entropy (NT-Xent) loss with a temperature of 0.1 and a batch size of 64. Models were optimised with Adam at a learning rate of 0.001 and trained until the training loss plateaued, indicating convergence.

**In contrast to SimCLR, which relies on large batch sizes to provide enough negative samples, MoCo-v2 maintains a dynamic memory queue of encoded samples that serves as a reservoir of negatives.** To ensure consistency between representations, MoCo-v2 uses a momentum encoder, which is a slowly evolving copy of the main (query) encoder, to generate key embeddings for the queue. **The momentum coefficient was linearly increased from 0.90 to 0.99 over training to improve stability. Training used the InfoNCE loss, Adam optimiser, learning rate of 0.001, temperature of 0.1 and queue size of 65,536.**

For both SimCLR and MoCo-v2, contrastive sampling was **patient-aware** each batch contained at most one heartbeat per patient. Positive pairs were constructed from two augmented views of the same heartbeat, while negatives were strictly cross-patient. In MoCo-v2, queued negatives originating from the same patient as the query were explicitly masked. This design prevents inadvertent patient identity leakage during contrastive pretraining.

### Augmentation ablation study using SimCLR

**To isolate the marginal effect of individual data augmentations, a controlled ablation in which exactly one augmentation was enabled at a time during SimCLR pretraining, in addition to a no-augmentation baseline. When enabled, an augmentation was applied deterministically to each view in the positive pair, while all other augmentations were disabled. Augmentations such as baseline drift (low-frequency wander), additive Gaussian noise, bounded global gain scaling (0.7-1.3x), short rigid temporal jitter** (±5 samples), mild global warping (± 20%), sinusoidal 50/60Hz power-line interference, single-lead dropout and single-lead polarity inversion.

Following self-supervised pretraining, encoder representations were fine-tuned on the Zhongshan-baseline dataset using a frozen-encoder linear probe and evaluated on a downstream Brugada syndrome classification task. The marginal effects of individual augmentations were quantified using repeated cross-validation across ten random seeds (0-9), with performance assessed using AUC and average precision (AP). Statistical significance of differences relative to the no-augmentation baseline was evaluated using paired t-tests across matched random seeds.

Table A summarises the effect of individual augmentation strategies on downstream performance across different pretraining datasets. When pretraining on PTB-XL, the no-augmentation baseline achieved the highest mean AUC, indicating strong overall ranking performance without enforced invariances. However, adding power-line hum during pretraining significantly improved average precision, yielding a substantial and statistically significant increase in AP without degrading AUC. This suggests that exposure to realistic narrow-band interference can improve minority-class retrieval performance under distribution shift. In contrast, most other augmentations, including baseline drift, Gaussian noise, gain scaling, and lead inversion, resulted in significant reductions in both AUC and AP, indicating that these perturbations may obscure diagnostically relevant morphology when treated as invariances.

For Zhongshan-pretrain and Imperial datasets, no-augmentation consistently achieved the best performance across both AUC and average precision. All single-augmentation variants led to degraded downstream performance, often with statistically significant decreases relative to the baseline. This pattern suggests that when the pretraining data distribution closely matches the downstream clinical setting, enforcing additional invariances through augmentation can be detrimental by removing subtle but informative signal characteristics. Overall, these results demonstrate that the effectiveness of ECG data augmentation in self-supervised learning is highly dependent on the pretraining dataset and the downstream task. Clinically motivated perturbations can be beneficial in certain transfer settings, but may also be harmful when they remove diagnostically relevant signal characteristics.

### **SMOTE oversampling**

To address class imbalance in the training data, we applied the Synthetic Minority Oversampling Technique (SMOTE) within each training fold, which generates artificial samples of the minority class, which in this case, is Brugada ECGs. Each sample was flattened to a one-dimensional vector (100 x 12 = 1200 features), and SMOTE was applied in this space. For a given minority class beat $x^{i}$, a synthetic beat $x^{synthetic}$ was generated by interpolating between $x^{i}$ and one of its $k$ = 5 nearest neighbours $x^{j}$, given as:

$$x^{synthetic}=x^{i}+c (x^{j}-x^{i})$$

where $c$ 𝜖 [0, 1] is a randomly sampled scalar for each feature. The resulting synthetic vectors were reshaped back to their original multilead shape (100 x 12).

In the 25%-75% split, SMOTE increased the number of individual beats for training from 413 to 1201; in the 80%-20% split, from 1333 to 3924. Importantly, the additional beats introduced by SMOTE are exclusively Brugada samples, aimed at balancing the class distribution.

**Table A. Effect of individual data augmentation strategies on downstream Brugada classification performance across pretraining datasets.**

| **SimCLR  Augmentation** | **AUC** | **Δ AUC** | **P-value** |  | **Average Precision** | **Δ Average Precision** | **P-value** |
| --- | --- | --- | --- | --- | --- | --- | --- |
| **PTB-XL** | | | | | | | |
| No Augmentation | **0.756 ± 0.023** | – | – |  | **0.489 ± 0.029** | **-** | – |
| Baseline Drift | 0.657 ± 0.034 | −0.099 ± 0.039 | 0.002 |  | 0.425 ± 0.033 | −0.064 ± 0.034 | 0.004 |
| Gain | 0.694 ± 0.025 | −0.062 ± 0.030 | 0.002 |  | 0.442 ± 0.030 | −0.047 ± 0.036 | 0.006 |
| Gaussian | 0.640 ± 0.035 | −0.116 ± 0.041 | 0.002 |  | 0.390 ± 0.034 | −0.099 ± 0.046 | 0.002 |
| **Hum** | **0.752 ± 0.021** | **−0.004 ± 0.029** | **1.000** |  | **0.548 ± 0.020** | **+0.059 ± 0.039** | **0.004** |
| Jitter | 0.724 ± 0.033 | −0.032 ± 0.037 | 0.004 |  | 0.494 ± 0.039 | +0.005 ± 0.045 | 0.769 |
| Lead Dropout | 0.729 ± 0.030 | −0.027 ± 0.034 | 0.027 |  | 0.506 ± 0.043 | +0.017 ± 0.058 | 0.322 |
| Lead Inversion | 0.686 ± 0.043 | −0.070 ± 0.046 | 0.006 |  | 0.438 ± 0.049 | −0.051 ± 0.053 | 0.027 |
| Time Warp | 0.736 ± 0.023 | −0.020 ± 0.036 | 0.131 |  | 0.498 ± 0.032 | +0.009 ± 0.048 | 0.922 |
| **Zhongshan-Pretrain** | | | | | | | |
| **No Augmentation** | **0.773 ± 0.021** | **–** | **–** |  | **0.532 ± 0.031** | **–** | **–** |
| Baseline Drift | 0.706 ± 0.024 | −0.067 ± 0.029 | 0.002 |  | 0.507 ± 0.024 | −0.025 ± 0.033 | 0.193 |
| Gain | 0.685 ± 0.036 | −0.088 ± 0.040 | 0.002 |  | 0.499 ± 0.035 | −0.033 ± 0.041 | 0.105 |
| Gaussian | 0.660 ± 0.048 | −0.113 ± 0.050 | 0.002 |  | 0.392 ± 0.041 | −0.140 ± 0.046 | 0.002 |
| **Hum** | 0.674 ± 0.023 | −0.099 ± 0.029 | 0.002 |  | 0.416 ± 0.026 | −0.116 ± 0.032 | 0.002 |
| Jitter | 0.646 ± 0.061 | −0.127 ± 0.064 | 0.002 |  | 0.448 ± 0.050 | −0.084 ± 0.056 | 0.006 |
| Lead Dropout | 0.693 ± 0.022 | −0.080 ± 0.028 | 0.002 |  | 0.432 ± 0.027 | −0.100 ± 0.033 | 0.002 |
| Lead Inversion | 0.597 ± 0.043 | −0.176 ± 0.048 | 0.002 |  | 0.347 ± 0.031 | −0.185 ± 0.036 | 0.002 |
| Time Warp | 0.614 ± 0.047 | −0.159 ± 0.051 | 0.002 |  | 0.368 ± 0.039 | −0.164 ± 0.044 | 0.002 |
| **Imperial** | | | | | | | |
| **No Augmentation** | **0.773 ± 0.021** | **–** | **–** |  | **0.531 ± 0.032** | **-** | **–** |
| Baseline Drift | 0.704 ± 0.025 | −0.070 ± 0.029 | 0.002 |  | 0.507 ± 0.027 | −0.024 ± 0.033 | 0.193 |
| Gain | 0.685 ± 0.037 | −0.088 ± 0.040 | 0.002 |  | 0.509 ± 0.039 | −0.022 ± 0.041 | 0.105 |
| Gaussian | 0.692 ± 0.022 | −0.081 ± 0.028 | 0.002 |  | 0.427 ± 0.031 | −0.104 ± 0.033 | 0.002 |
| **Hum** | 0.674 ± 0.025 | −0.100 ± 0.029 | 0.002 |  | 0.416 ± 0.029 | −0.115 ± 0.032 | 0.002 |
| Jitter | 0.654 ± 0.051 | −0.120 ± 0.050 | 0.002 |  | 0.402 ± 0.046 | −0.129 ± 0.046 | 0.002 |
| Lead Dropout | 0.636 ± 0.065 | −0.138 ± 0.064 | 0.002 |  | 0.435 ± 0.061 | −0.096 ± 0.056 | 0.006 |
| Lead Inversion | 0.614 ± 0.047 | −0.160 ± 0.051 | 0.002 |  | 0.362 ± 0.043 | −0.169 ± 0.044 | 0.002 |
| Time Warp | 0.593 ± 0.038 | −0.180 ± 0.048 | 0.002 |  | 0.346 ± 0.030 | −0.185 ± 0.036 | 0.002 |

Note: AUC - Area under the curve of receiver operating characteristic curve; Δ – change from “no augmentation”. Tested models used the SimCLR framework.

**Table B. Averaged performance metrics (mean ± SD) after 8-fold cross-validation using a 25%-75% training-test split.**

| **Pretraining**  **method** | **Pretraining**  **data** | **SMOTE** | **Accuracy** | **F1-score** | **AUC** | **Sensitivity** | **Specificity** | **NPV** | **PPV** | **Average precision** |
| --- | --- | --- | --- | --- | --- | --- | --- | --- | --- | --- |
| **Baseline** | | | | | | | | | | |
| N | N | N | 0.921 ± 0.030  [0.896, 0.947] | 0.810 ± 0.092  [0.733, 0.887] | 0.946 ± 0.061  [0.895, 0.997] | 0.727 ± 0.171  [0.584, 0.870] | 0.983 ± 0.030  [0.958, 1.000] | 0.920 ± 0.047  [0.881, 0.959] | 0.954 ± 0.070  [0.896, 1.000] | 0.912± 0.099  [0.829, 0.995] |
| N | N | Y | 0.877 ± 0.088  [0.804, 0.950] | 0.850 ± 0.124  [0.746, 0.953] | 0.958 ± 0.070  [0.899, 1.000] | 0.764 ± 0.182  [0.611, 0.916] | 0.990 ± 0.016  [0.977, 1.000] | 0.823 ± 0.116  [0.726, 0.920] | 0.989 ± 0.016  [0.975, 1.000] | 0.960 ± 0.058  [0.912, 1.000] |
| **Supervised pretraining** | | | | | | | | | | |
| Supervised | PTB-XL | N | 0.913 ± 0.047  [0.874, 0.952] | 0.785 ± 0.147  [0.662, 0.907] | 0.938 ± 0.090  [0.863, 1.000] | 0.714 ± 0.186  [0.558, 0.869] | 0.976 ± 0.031  [0.950, 1.000] | 0.917 ± 0.047  [0.878, 0.956] | 0.898 ± 0.127  [0.792, 1.000] | 0.891 ± 0.129  [0.784, 0.999] |
| Supervised | PTB-XL | Y | 0.847 ± 0.091  [0.772, 0.923] | 0.822 ± 0.123  [0.719, 0.924] | 0.969 ± 0.109  [0.878, 1.000] | 0.730 ± 0.172  [0.586, 0.874] | 0.982 ± 0.015  [0.969, 0.995] | 0.787 ± 0.113  [0.692, 0.881] | 0.970 ± 0.026  [0.948, 0.991] | 0.949 ± 0.070  [0.891, 1.000] |
| Supervised | Zhongshan-pretrain | N | 0.932 ± 0.036  [0.902, 0.962] | 0.833 ± 0.120  [0.733, 0.934] | 0.979 ± 0.017  [0.965, 0.993] | 0.765 ± 0.140  [0.658, 0.881] | 0.983 ± 0.020  [0.966, 1.000] | 0.932 ± 0.034  [0.904, 0.960] | 0.924 ± 0.121  [0.823, 1.000] | 0.933 ± 0.078  [0.868, 0.998] |
| Supervised | Zhongshan-pretrain | Y | 0.883 ± 0.053  [0.839, 0.927] | 0.866 ± 0.064  [0.813, 0.920] | 0.980 ± 0.022  [0.962, 0.998] | 0.779 ± 0.108  [0.690, 0.869] | 0.987 ± 0.016  [0.974, 1.000] | 0.824 ± 0.079  [0.758, 0.889] | 0.984 ± 0.020  [0.967, 1.000] | 0.978 ± 0.024  [0.958, 0.998] |
| **Self-supervised pretraining** | | | | | | | | | | |
| SimCLR | PTB-XL | N | 0.784 ± 0.054  [0.739, 0.830] | 0.363 ± 0.107  [0.273, 0.452] | 0.782 ± 0.099  [0.699, 0.865] | 0.249 ± 0.093  [0.171, 0.374] | 0.948 ± 0.061  [0.897, 0.999] | 0.932 ± 0.058  [0.884, 0.980] | 0.843 ± 0.134  [0.731, 0.955] | 0.917 ± 0.051  [0.874, 0.959] |
| SimCLR | PTB-XL | Y | 0.762 ± 0.095  [0.683, 0.842] | 0.758 ± 0.128  [0.651, 0.865] | 0.832 ± 0.108  [0.742, 0.922] | 0.710 ± 0.169  [0.569, 0.852] | 0.810 ± 0.090  [0.736, 0.885] | 0.742 ± 0.105  [0.654, 0.830] | 0.829 ± 0.104  [0.742, 0.916] | 0.852 ± 0.104  [0.766, 0.939] |
| SimCLR | Imperial | N | 0.929 ± 0.032  [0.902, 0.955] | 0.848 ± 0.117  [0.750, 0.946] | 0.947 ± 0.088  [0.873, 1.000] | 0.848 ± 0.152  [0.721, 0.975] | 0.957 ± 0.039  [0.925, 0.990] | 0.950 ± 0.046  [0.911, 0.988] | 0.866 ± 0.161  [0.732, 1.000] | 0.893 ± 0.132  [0.783, 1.000] |
| SimCLR | Imperial | Y | 0.930 ± 0.049  [0.888, 0.971] | 0.925 ± 0.054  [0.880, 0.971] | 0.976 ± 0.027  [0.954, 0.999] | 0.891 ± 0.078  [0.826, 0.957] | 0.968 ± 0.033  [0.940, 0.995] | 0.902 ± 0.065  [0.848, 0.957] | 0.964 ± 0.036  [0.934, 0.995] | 0.980 ± 0.019  [0.965, 0.996] |
| SimCLR | Zhongshan-pretrain | N | 0.769 ± 0.047  [0.730, 0.808] | 0.295 ± 0.149  [0.092, 0.665] | 0.655 ± 0.145  [0.533, 0.776] | 0.185 ± 0.106  [0.125, 0.273] | 0.970 ± 0.049  [0.930, 1.000] | 0.786 ± 0.054  [0.741, 0.831] | 0.274 ± 0.185  [0.016, 0.568] | 0.473 ± 0.130  [0.364, 0.582] |
| SimCLR | Zhongshan-pretrain | Y | 0.762 ± 0.095  [0.683, 0.842] | 0.758 ± 0.128  [0.651, 0.865] | 0.832 ± 0.108  [0.742, 0.922] | 0.710 ± 0.169  [0.569, 0.852] | 0.810 ± 0.090  [0.736, 0.885] | 0.742 ± 0.105  [0.654, 0.830] | 0.829 ± 0.104  [0.742, 0.916] | 0.852 ± 0.104  [0.921, 0.939] |
| MoCo-V2 | PTB-XL | N | 0.906 ± 0.044  [0.870, 0.943] | 0.798 ± 0.124  [0.694, 0.901] | 0.964 ± 0.111  [0.872, 1.000] | 0.716 ± 0.126  [0.610, 0.821] | 0.980 ± 0.051  [0.956, 1.000] | 0.914 ± 0.036  [0.884, 0.944] | 0.933 ± 0.179  [0.991, 1.000] | 0.928 ± 0.113  [0.833, 1.000] |
| MoCo-V2 | PTB-XL | Y | 0.903 ± 0.040  [0.870, 0.937] | 0.900 ± 0.043  [0.864, 0.936] | 0.966 ± 0.019  [0.950, 0.982] | 0.877 ± 0.750  [0.814, 0.940] | 0.930 ± 0.055  [0.884, 0.976] | 0.887 ± 0.061  [0.836, 0.939] | 0.929 ± 0.052  [0.886, 0.973] | 0.971 ± 0.017  [0.957, 0.985] |
| MoCo-V2 | Imperial | N | 0.868 ± 0.055  [0.822, 0.914] | 0.724 ± 0.134  [0.612, 0.836] | 0.912 ± 0.075  [0.849, 0.975] | 0.713 ± 0.119  [0.614, 0.812] | 0.922 ± 0.059  [0.873, 0.971] | 0.906 ± 0.041  [0.872, 0.940] | 0.757 ± 0.186  [0.602, 0.913] | 0.831 ± 0.130  [0.722, 0.939] |
| MoCo-V2 | Imperial | Y | 0.853 ± 0.089  [0.779, 0.927] | 0.880 ± 0.112  [0.786, 0.973] | 0.945 ± 0.086  [0.873, 1.000] | 0.861 ± 0.143  [0.741, 0.980] | 0.918 ± 0.057  [0.871, 0.966] | 0.816 ± 0.097  [0.735, 0.897] | 0.902 ± 0.074  [0.840, 0.963] | 0.967 ± 0.073  [0.890, 1.000] |
| MoCo-V2 | Zhongshan-pretrain | N | 0.842 ± 0.047  [0.802, 0.881] | 0.645 ± 0.164  [0.509, 0.782] | 0.841 ± 0.122  [0.739, 0.943] | 0.571 ± 0.170  [0.429, 0.713] | 0.943 ± 0.049  [0.903, 0.984] | 0.870 ± 0.048  [0.829, 0.910] | 0.778 ± 0.190  [0.619, 0.936] | 0.740 ± 0.187  [0.583, 0.897] |
| MoCo-V2 | Zhongshan-pretrain | Y | 0.774 ± 0.093  [0.696, 0.851] | 0.748 ± 0.125  [0.643, 0.853] | 0.851 ± 0.114  [0.756, 0.947] | 0.706 ± 0.172  [0.563, 0.850] | 0.842 ± 0.051  [0.799, 0.884] | 0.754 ± 0.112  [0.661, 0.848] | 0.811 ± 0.063  [0.759, 0.864] | 0.866 ± 0.093  [0.788, 0.943] |

Note: Y- Yes; N-No; AUC - Area under the curve of receiver operating characteristic curve; NPV – Negative predictive value; PPV – Positive predictive value. Data are presented as mean ± standard deviation and [5^th^ percentile, 95^th^ percentile]

**Table C. Paired bootstrap comparison of model performance across train-test splits between the baseline model and two pretrained alternatives.**

| **Metrics** | **Accuracy** | **F1-score** | **AUC** | **Sensitivity** | **Specificity** | **NPV** | **PPV** | **Average precision** | **Brier score** |  |
| --- | --- | --- | --- | --- | --- | --- | --- | --- | --- | --- |
| **Baseline vs. Supervised Zhongshan, at 25-75% train-test split** | | | | | | | | | | |
| Mean Difference | +0.032 | +0.071 | +0.019 | +0.095 | +0.010 | +0.031 | +0.038 | +0.034 | -0.032 |  |
| 95^th^ Confidence Interval [5^th^, 95^th^] | [0.027, 0.038] | [0.059, 0.084] | [0.013, 0.025] | [0.077, 0.113] | [0.006, 0.015] | [0.025, 0.036] | [0.023, 0.052] | [0.025, 0.043] | [-0.037,  -0.027] |  |
| P-value | **< 0.001** | **< 0.001** | **< 0.001** | **< 0.001** | **< 0.001** | **< 0.001** | **< 0.001** | **< 0.001** | **< 0.001** |  |
| **Baseline vs. SimCLR PTB-XL, at 25-75% train-test split** | | | | | | | | | | |
| Mean Difference | +0.005 | +0.018 | -0.006 | +0.060 | -0.015 | -0.038 | +0.018 | -0.023 | -0.009 |  |
| 95^th^ Confidence Interval [5^th^, 95^th^] | [-0.002, 0.010] | [0.005, 0.030] | [-0.012, 0.001] | [0.042, 0.077] | [-0.020, -0.010] | [-0.053, -0.023] | [0.012, 0.023] | [-0.033, -0.013] | [-0.014,  -0.004] |  |
| P-value | 0.188 | **0.002** | 0.064 | **< 0.001** | **< 0.001** | **< 0.001** | **< 0.001** | **< 0.001** | **< 0.001** |  |
| **Baseline vs. Supervised Zhongshan, at 80-20% train-test split** | | | | | | | | | | |
| Mean Difference | +0.017 | +0.034 | -0.006 | +0.051 | +0.005 | +0.017 | +0.016 | -0.005 | -0.014 |  |
| 95^th^ Confidence Interval [5^th^, 95^th^] | [0.009, 0.024] | [0.018, 0.049] | [-0.015, 0.002] | [0.026, 0.075] | [-0.001, -0.011] | [0.009, 0.025] | [0.000, 0.032] | [-0.015, 0.004] | [-0.021, -0.008] |  |
| P-value | **< 0.001** | **< 0.001** | 0.210 | **< 0.001** | 0.140 | **< 0.001** | 0.052 | 0.383 | **< 0.001** |  |
| **Baseline vs. SimCLR PTB-XL, at 80-20% train-test split** | | | | | | | | | | |
| Mean Difference | +0.009 | +0.018 | +0.002 | +0.019 | +0.005 | +0.007 | +0.017 | +0.006 | -0.010 |  |
| 95^th^ Confidence Interval [5^th^, 95^th^] | [0.002, 0.016] | [0.004, 0.033] | [-0.002, 0.005] | [-0.002, 0.043] | [0.000, 0.011] | [-0.001, 0.014] | [0.001, 0.032] | [0.001, 0.012] | [-0.016,  -0.005] |  |
| P-value | 0.018 | 0.018 | 0.426 | 0.121 | 0.076 | 0.064 | 0.023 | 0.112 | **< 0.001** |  |

**Table D. Brugada classification model performance using the 80%-20% training-test split.**

| **Pretraining**  **method** | **Pretraining**  **data** | **SMOTE** | **Accuracy (%)** | **F1-score** | **AUC** | **Sensitivity** | **Specificity** | **NPV** | **PPV** | **Average Precision** | **Brier score** |
| --- | --- | --- | --- | --- | --- | --- | --- | --- | --- | --- | --- |
| **Baseline** | | | | | | | | | | |  |
| N | N | N | 96.6 | 0.932 | 0.993 | 0.911 | 0.985 | 0.970 | 0.955 | 0.983 | 0.030 |
| N | N | Y | 97.2 | 0.944 | 0.994 | 0.939 | 0.983 | 0.979 | 0.950 | 0.986 | 0.024 |
| **Supervised pretraining** | | | | | | | | | | |  |
| Supervised | PTB-XL | N | 98.1 * ▲ | 0.962 | 0.998 | 0.951 | 0.991 | 0.983 | 0.974 | 0.994 | 0.017 |
| Supervised | PTB-XL | Y | 94.8 ^†,‡^ ▼ | 0.904 | 0.993 ^‡^ ▼ | 0.961 | 0.944 | 0.986 | 0.854 | 0.980 | 0.044 |
| Supervised | Zhongshan-pretrain | N | 98.3 * ▲ | 0.966 | 0.986 | 0.963 | 0.990 | 0.987 | 0.970 | 0.978 | 0.016 |
| Supervised | Zhongshan-pretrain | Y | 98.3 ^†^ ▲ | 0.966 | 0.999 ^‡^ ▲ | 0.953 | 0.993 | 0.984 | 0.980 | 0.983 | 0.014 |
| **Self-supervised pretraining** | | | | | | | | | | |  |
| SimCLR | PTB-XL | N | 97.5 | 0.951 | 0.994 | 0.931 | 0.990 | 0.977 | 0.971 | 0.989 | 0.019 |
| SimCLR | PTB-XL | Y | 97.6 | 0.952 | 0.994 | 0.929 | 0.992 | 0.976 | 0.975 | 0.988 | 0.023 |
| SimCLR | Imperial | N | 96.7 | 0.935 | 0.992 | 0.923 | 0.982 | 0.974 | 0.947 | 0.981 | 0.028 |
| SimCLR | Imperial | Y | 96.7 | 0.934 | 0.992 | 0.917 | 0.984 | 0.972 | 0.951 | 0.980 | 0.030 |
| SimCLR | Zhongshan-pretrain | N | 97.0 | 0.938 | 0.995 | 0.895 | 0.995 | 0.965 | 0.985 | 0.988 | 0.024 |
| SimCLR | Zhongshan-pretrain | Y | 97.3 | 0.946 | 0.995 | 0.913 | 0.994 | 0.971 | 0.981 | 0.988 | 0.023 |
| MoCo-V2 | PTB-XL | N | 97.0 | 0.939 | 0.987 | 0.909 | 0.990 | 0.969 | 0.971 | 0.972 | 0.027 |
| MoCo-V2 | PTB-XL | Y | 97.5 | 0.950 | 0.983 | 0.935 | 0.988 | 0.978 | 0.965 | 0.967 | 0.023 |
| MoCo-V2 | Imperial | N | 95.7 *▼ | 0.914 | 0.982 *▼ | 0.903 | 0.975 | 0.967 | 0.925 | 0.963 | 0.041 |
| MoCo-V2 | Imperial | Y | 95.4 | 0.907 | 0.985 | 0.874 | 0.982 | 0.958 | 0.943 | 0.968 | 0.042 |
| MoCo-V2 | Zhongshan-pretrain | N | 97.3 | 0.946 | 0.996 | 0.933 | 0.986 | 0.977 | 0.959 | 0.989 | 0.022 |
| MoCo-V2 | Zhongshan-pretrain | Y | 96.5 | 0.931 | 0.993 | 0.925 | 0.979 | 0.974 | 0.938 | 0.985 | 0.027 |

Note: Y- Yes; N-No; SMOTE - synthetic minority oversampling technique; AUC - Area under the receiver operating characteristic curve; NPV – Negative predictive value; PPV – Positive predictive value.

** P<0.002 comparing each model (no SMOTE) against the baseline (no SMOTE).*

^†^ *P<0.002 comparing each model (SMOTE) against the baseline (SMOTE).*

^‡^ *P<0.002 comparing each model (no SMOTE) with the same model (SMOTE).*

In the “AUC” column, significance was assessed with the Holm-corrected DeLong test. In the “Acc. (%)” column, significance was assessed with the Holm-corrected McNemar test. *▲ indicates improvement, ▼ indicates worsening.*

**Table E. Averaged performance metrics (mean ± SD) after 8-fold cross-validation using 80%-20% training-test split**

| **Pretraining**  **method** | **Pretraining**  **data** | **SMOTE** | **Accuracy** | **F1-score** | **AUC** | **Sensitivity** | **Specificity** | **NPV** | **PPV** | **Average precision** |
| --- | --- | --- | --- | --- | --- | --- | --- | --- | --- | --- |
| **Baseline** | | | | | | | | | | |
| N | N | N | 0.968 ± 0.016  [0.954, 0.981] | 0.964 ± 0.018  [0.949, 0.979] | 0.994 ± 0.055  [0.990, 0.999] | 0.949 ± 0.032  [0.922, 0.975] | 0.984 ± 0.012  [0.973, 0.994] | 0.959 ± 0.025  [0.938, 0.980] | 0.980 ± 0.015  [0.968, 0.992] | 0.992 ± 0.007  [0.987, 0.998] |
| N | N | Y | 0.958 ± 0.014  [0.947, 0.970] | 0.958 ± 0.014  [0.947, 0.970] | 0.994 ± 0.004  [0.990, 0.997] | 0.951 ± 0.038  [0.919, 0.983] | 0.967 ± 0.035  [0.938, 0.996] | 0.954 ± 0.035  [0.924, 0.983] | 0.968 ± 0.031  [0.942, 0.994] | 0.993 ± 0.005  [0.989, 0.997] |
| **Supervised pretraining** | | | | | | | | | | |
| Supervised | PTB-XL | N | 0.964 ± 0.026  [0.942, 0.985] | 0.959 ± 0.030  [0.934, 0.984] | 0.992 ± 0.010  [0.983, 1.000] | 0.948 ± 0.058  [0.900, 0.996] | 0.976 ± 0.028  [0.953, 1.000] | 0.960 ± 0.043  [0.924, 0.996] | 0.974 ± 0.028  [0.950, 0.997] | 0.988 ± 0.020  [0.972, 1.000] |
| Supervised | PTB-XL | Y | 0.962 ± 0.029  [0.938, 0.986] | 0.960 ± 0.031  [0.934, 0.987] | 0.991 ± 0.010  [0.983, 0.999] | 0.950 ± 0.059  [0.901, 0.999] | 0.973 ± 0.024  [0.953, 0.993] | 0.954 ± 0.050  [0.912, 0.996] | 0.973 ± 0.023  [0.954, 0.993] | 0.989 ± 0.015  [0.976, 1.000] |
| Supervised | Zhongshan-pretrain | N | 0.972 ± 0.018  [0.957, 0.987] | 0.969 ± 0.020  [0.952, 0.986] | 0.998 ± 0.002  [0.996, 0.999] | 0.959 ± 0.040  [0.925, 0.992] | 0.983 ± 0.017  [0.968, 0.997] | 0.968 ± 0.030  [0.942, 0.993] | 0.980 ± 0.019  [0.964, 0.996] | 0.998 ± 0.002  [0.996, 0.999] |
| Supervised | Zhongshan-pretrain | Y | 0.972 ± 0.016  [0.959, 0.985] | 0.971 ± 0.016  [0.958, 0.985] | 0.998 ± 0.002  [0.996, 0.999] | 0.958 ± 0.032  [0.931, 0.985] | 0.986 ± 0.014  [0.974, 0.998] | 0.960 ± 0.030  [0.935, 0.985] | 0.986 ± 0.014  [0.974, 0.997] | 0.998 ± 0.002  [0.996, 0.999] |
| **Self-supervised pretraining** | | | | | | | | | | |
| SimCLR | PTB-XL | N | 0.982 ± 0.015  [0.970, 0.995] | 0.981 ± 0.017  [0.967, 0.995] | 0.996 ± 0.008  [0.989, 1.000] | 0.976 ± 0.025  [0.954, 0.997] | 0.988 ± 0.021  [0.970, 1.000] | 0.980 ± 0.020  [0.963, 0.997] | 0.986 ± 0.022  [0.968, 1.000] | 0.993 ± 0.017  [0.979, 1.000] |
| SimCLR | PTB-XL | Y | 0.981 ± 0.018  [0.965, 0.996] | 0.980 ± 0.019  [0.965, 0.996] | 0.997 ± 0.005  [0.992, 1.000] | 0.975 ± 0.030  [0.950, 1.000] | 0.987 ± 0.020  [0.970, 1.000] | 0.976 ± 0.028  [0.953, 0.999] | 0.987 ± 0.019  [0.971, 1.000] | 0.995 ± 0.009  [0.988, 1.000] |
| SimCLR | Imperial | N | 0.929 ± 0.044  [0.892, 0.965] | 0.853 ± 0.096  [0.773, 0.934] | 0.964 ± 0.043  [0.929, 1.000] | 0.813 ± 0.129  [0.705, 0.921] | 0.971 ± 0.020  [0.954, 0.987] | 0.937 ± 0.042  [0.901, 0.972] | 0.904 ± 0.073  [0.843, 0.965] | 0.913 ± 0.092  [0.836, 0.989] |
| SimCLR | Imperial | Y | 0.968 ± 0.011  [0.959, 0.977] | 0.968 ± 0.011  [0.958, 0.977] | 0.996 ± 0.002  [0.994, 0.997] | 0.959 ± 0.026  [0.938, 0.981] | 0.977 ± 0.013  [0.966, 0.988] | 0.961 ± 0.024  [0.941, 0.981] | 0.964 ± 0.036  [0.934, 0.995] | 0.980 ± 0.019  [0.965, 0.996] |
| SimCLR | Zhongshan-pretrain | N | 0.937 ± 0.040  [0.903, 0.970] | 0.870 ± 0.096  [0.789, 0.950] | 0.969 ± 0.035  [0.940, 0.998] | 0.842 ± 0.132  [0.732, 0.952] | 0.970 ± 0.018  [0.955, 0.985] | 0.947 ± 0.040  [0.913, 0.980] | 0.907 ± 0.059  [0.857, 0.956] | 0.933 ± 0.067  [0.877, 0.989] |
| SimCLR | Zhongshan-pretrain | Y | 0.916 ± 0.058  [0.868, 0.965] | 0.910 ± 0.069  [0.852, 0.967] | 0.970 ± 0.034  [0.941, 0.998] | 0.874 ± 0.106  [0.786, 0.963] | 0.959 ± 0.026  [0.937, 0.981] | 0.890 ± 0.080  [0.823, 0.958] | 0.954 ± 0.030  [0.929, 0.979] | 0.970 ± 0.032  [0.944, 0.997] |
| MoCo-V2 | PTB-XL | N | 0.942 ± 0.036  [0.912, 0.971] | 0.879 ± 0.093  [0.801, 0.957] | 0.970 ± 0.030  [0.945, 0.995] | 0.869 ± 0.141  [0.751, 0.987] | 0.967 ± 0.009  [0.959, 0.974] | 0.957 ± 0.041  [0.922, 0.991] | 0.900 ± 0.029  [0.875, 0.925] | 0.923 ± 0.058  [0.874, 0.971] |
| MoCo-V2 | PTB-XL | Y | 0.952 ± 0.013  [0.942, 0.963] | 0.951 ± 0.013  [0.940, 0.962] | 0.992 ± 0.005  [0.988, 0.996] | 0.936 ± 0.034  [0.907, 0.964] | 0.969 ± 0.029  [0.945, 0.993] | 0.939 ± 0.029  [0.915, 0.963] | 0.969 ± 0.028  [0.946, 0.992] | 0.991 ± 0.006  [0.986, 0.996] |
| MoCo-V2 | Imperial | N | 0.940 ± 0.031  [0.914, 0.966] | 0.877 ± 0.073  [0.816, 0.938] | 0.973 ± 0.027  [0.950, 0.995] | 0.844 ± 0.119  [0.744, 0.943] | 0.974 ± 0.015  [0.962, 0.987] | 0.947 ± 0.037  [0.917, 0.978] | 0.922 ± 0.038  [0.890, 0.954] | 0.941 ± 0.050  [0.899, 0.982] |
| MoCo-V2 | Imperial | Y | 0.946 ± 0.051  [0.904, 0.989] | 0.943 ± 0.056  [0.897, 0.990] | 0.973 ± 0.033  [0.945, 1.000] | 0.921 ± 0.082  [0.852, 0.989] | 0.972 ± 0.026  [0.950, 0.994] | 0.928 ± 0.069  [0.871, 0.986] | 0.970 ± 0.030  [0.944, 0.995] | 0.980 ± 0.021  [0.963, 0.997] |
| MoCo-V2 | Zhongshan-pretrain | N | 0.939 ± 0.043  [0.903, 0.974] | 0.877 ± 0.094  [0.798, 0.956] | 0.974 ± 0.029  [0.949, 0.998] | 0.858 ± 0.120  [0.758, 0.958] | 0.967 ± 0.026  [0.946, 0.989] | 0.952 ± 0.037  [0.921, 0.983] | 0.902 ± 0.074  [0.840, 0.963] | 0.935 ± 0.074  [0.874, 0.997] |
| MoCo-V2 | Zhongshan-pretrain | Y | 0.920 ± 0.052  [0.877, 0.963] | 0.915 ± 0.059  [0.866, 0.964] | 0.974 ± 0.023  [0.955, 0.993] | 0.886 ± 0.091  [0.810, 0.962] | 0.954 ± 0.039  [0.921, 0.987] | 0.898 ± 0.072  [0.838, 0.959] | 0.951 ± 0.039  [0.918, 0.983] | 0.971 ± 0.028  [0.947, 0.994] |

Note: Y- Yes; N-No; AUC - Area under the curve of the receiver operating characteristic curve; NPV – Negative predictive value; PPV – Positive predictive value. Data are presented as mean ± standard deviation and [5^th^ percentile, 95^th^ percentile]

**Table F. Comparison of model training time for each Brugada classification model trained on the 25%-75% training-test split.**

| **Pretraining method** | **Pretraining data** | **Number of training samples** | **Pretraining training time (seconds)** | **Training or fine-tuning training time (seconds)** | **Total training time  (% of baseline)** |
| --- | --- | --- | --- | --- | --- |
| **Baseline** | | | | | |
| N | N | 5257 | - | 470 | 100% |
| **Supervised pretraining** | | | | | |
| Supervised | PTB-XL | 227,199 | 17,555 | 496 | 3,841% |
| Supervised | Zhongshan-pretrain | 180,575 | 1,175 | 548 | 367% |
| **Self-supervised pretraining** | | | | | |
| SimCLR | PTB-XL | 227,199 | 35,786 | 1105 | 7,849% |
| SimCLR | Imperial | 14,216 | 2,255 | 566 | 600% |
| SimCLR | Zhongshan-pretrain | 180,575 | 28,593 | 1105 | 6,318% |
| MoCo-V2 | PTB-XL | 227,199 | 90,237 | 1270 | 194,697% |
| MoCo-V2 | Imperial | 14,216 | 7066 | 592 | 1,629% |
| MoCo-V2 | Zhongshan-pretrain | 180,575 | 45,123 | 1427 | 9,904% |

**Table G. Comparison of model training time for each Brugada classification model trained on the 80%-20% training-test split.**

| **Pretraining method** | **Pretraining data** | **Number of training samples** | **Pretraining training time (seconds)** | **Training or fine-tuning training time (seconds)** | **Total training time  (% of baseline)** |
| --- | --- | --- | --- | --- | --- |
| **Baseline** | | | | | |
| N | N | 2364 | - | 622 | 100% |
| **Supervised pretraining** | | | | | |
| Supervised | PTB-XL | 227,199 | 17,555 | 1123 | 3,002% |
| Supervised | Zhongshan-pretrain | 180,575 | 1,175 | 1624 | 450% |
| **Self-supervised pretraining** | | | | | |
| SimCLR | PTB-XL | 227,199 | 35,786 | 1797 | 6,042% |
| SimCLR | Imperial | 14,216 | 2,255 | 829 | 496% |
| SimCLR | Zhongshan-pretrain | 180,575 | 28,593 | 2885 | 5,061% |
| MoCo-V2 | PTB-XL | 227,199 | 90,237 | 2695 | 149,409% |
| MoCo-V2 | Imperial | 14,216 | 7066 | 898 | 1,280% |
| MoCo-V2 | Zhongshan-pretrain | 180,575 | 45,123 | 2522 | 7,660% |

**Fig A. Learning curve analysis comparing baseline model and supervised pretrained model on Zhongshan-pretrain dataset**


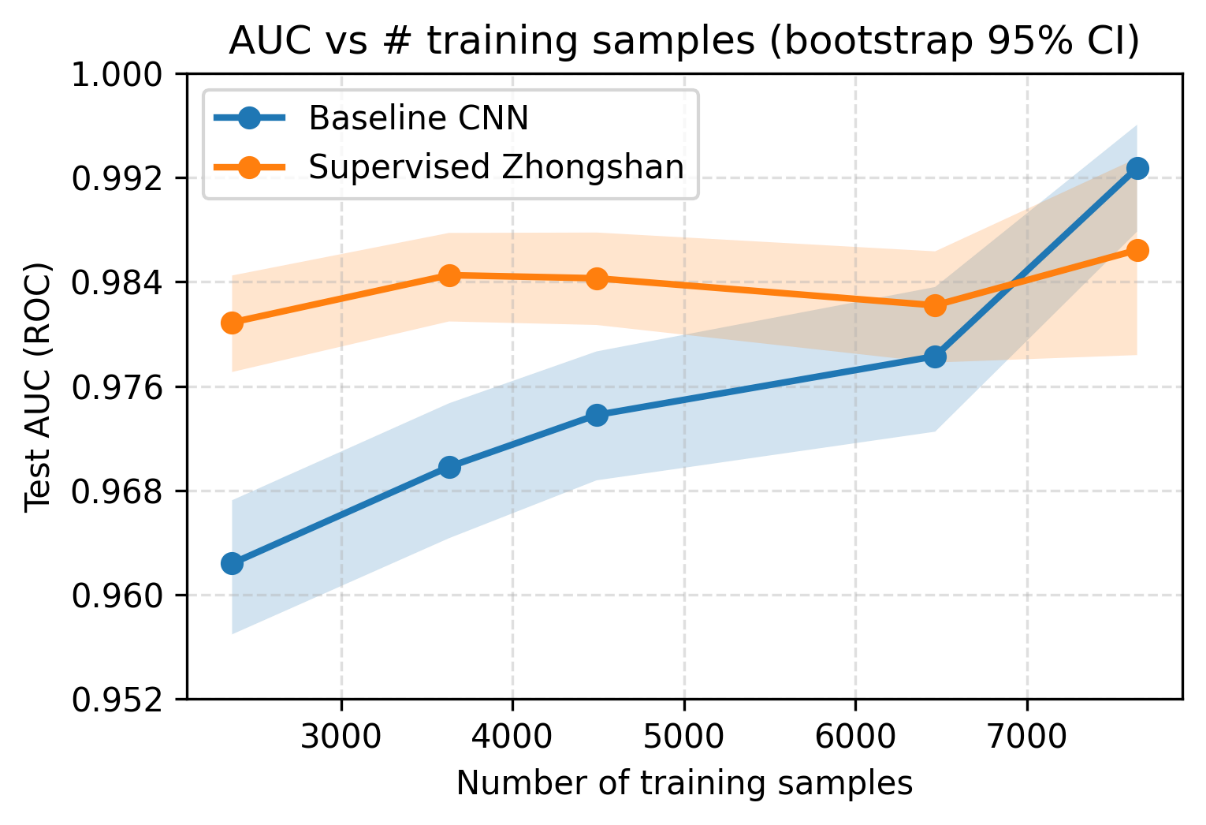


Learning curve showing test-set AUC as a function of the number of labelled training samples. The shaded bands show non-parametric bootstrap 95% confidence intervals for each model’s AUC (2000 resamples of the test set with replacement per split and per model).

**Fig B. Additional explainability assessments of the baseline and two pretrained models.**

| 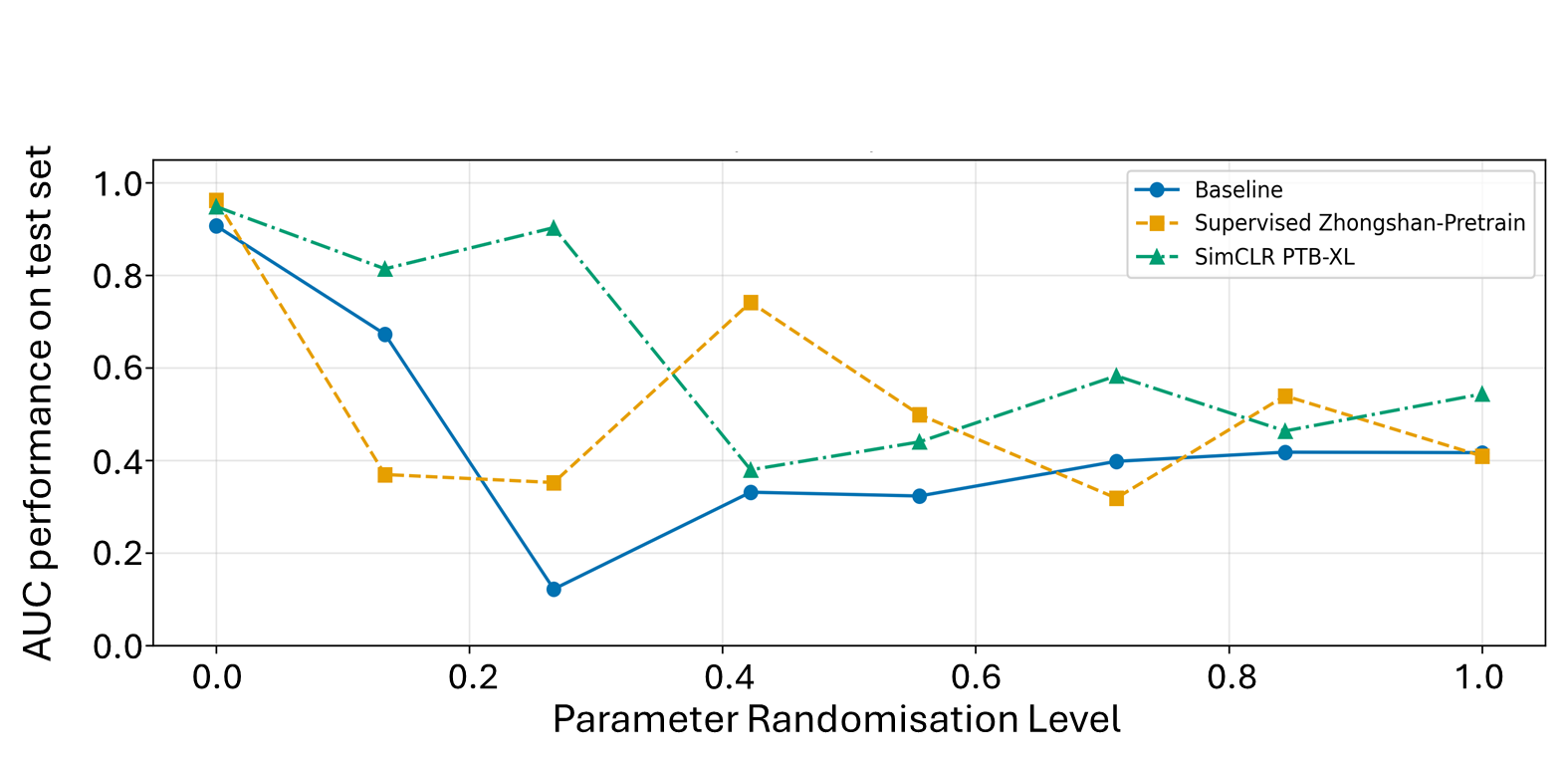 | 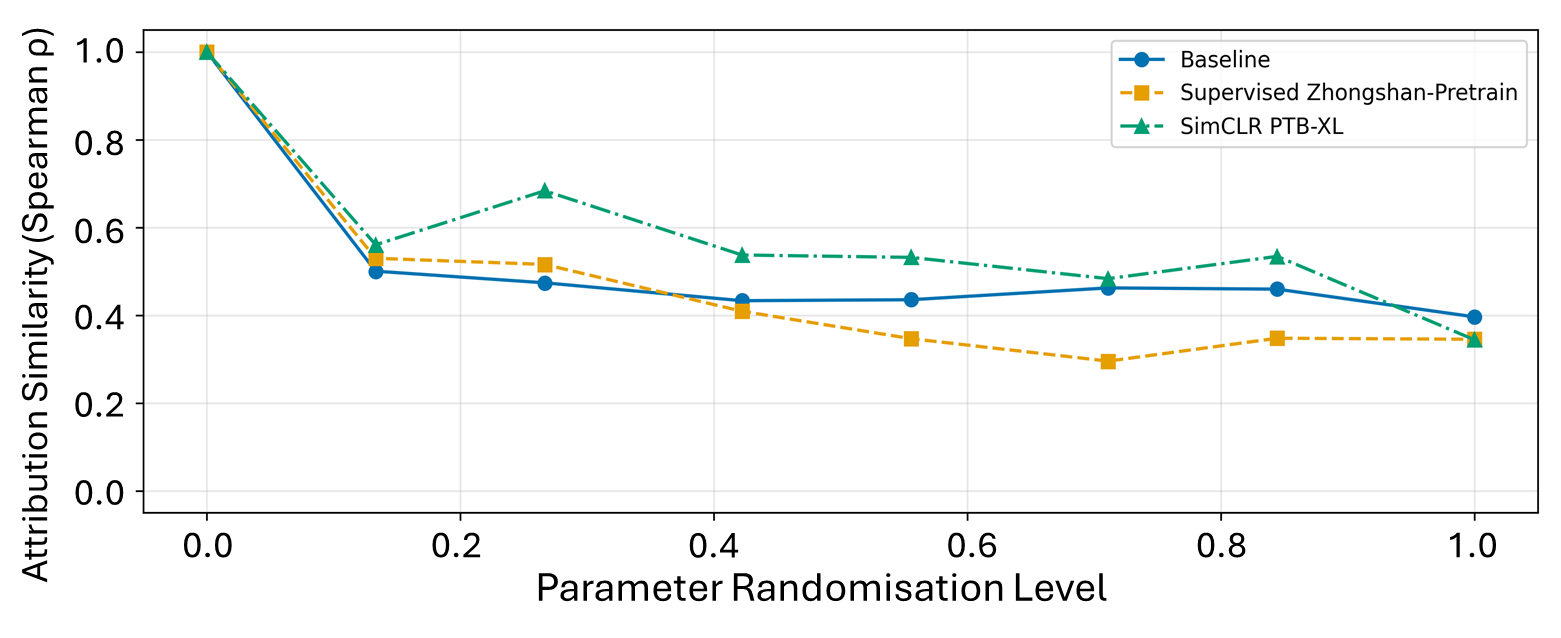 |
| --- | --- |
| (A) | (B) |
| 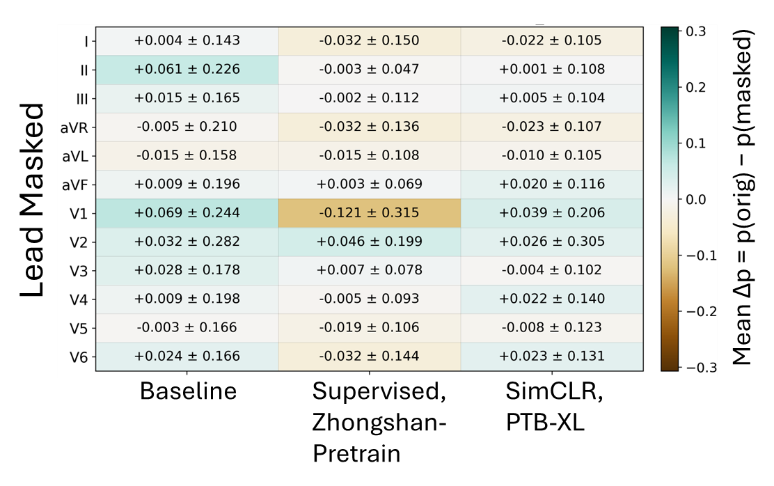 | |
| (C) | |

(A) Model discrimination (AUC on the test set) as a function of progressive parameter randomisation, following Adebayo et al. Randomising increasing fractions of network parameters degrades performance toward chance, confirming dependence on learned representations. (B) Attribution similarity (Spearman correlation of SHAP maps) between the original model and partially randomised models. Similarity decreases monotonically with increasing randomisation, indicating that attributions are not invariant to model parameters and are therefore model-dependent rather than input artifacts. (C) Lead ablation analysis showing mean change in predicted probability after masking individual ECG leads (Δ*p* = *p*_ORIG_ – *p*_masked_). Positive values indicate leads that support Brugada prediction; negative values indicate leads whose features suppress Brugada probability in the full model.

**Fig C. SHAP-based explanation overlays for Brugada ECG beats.**
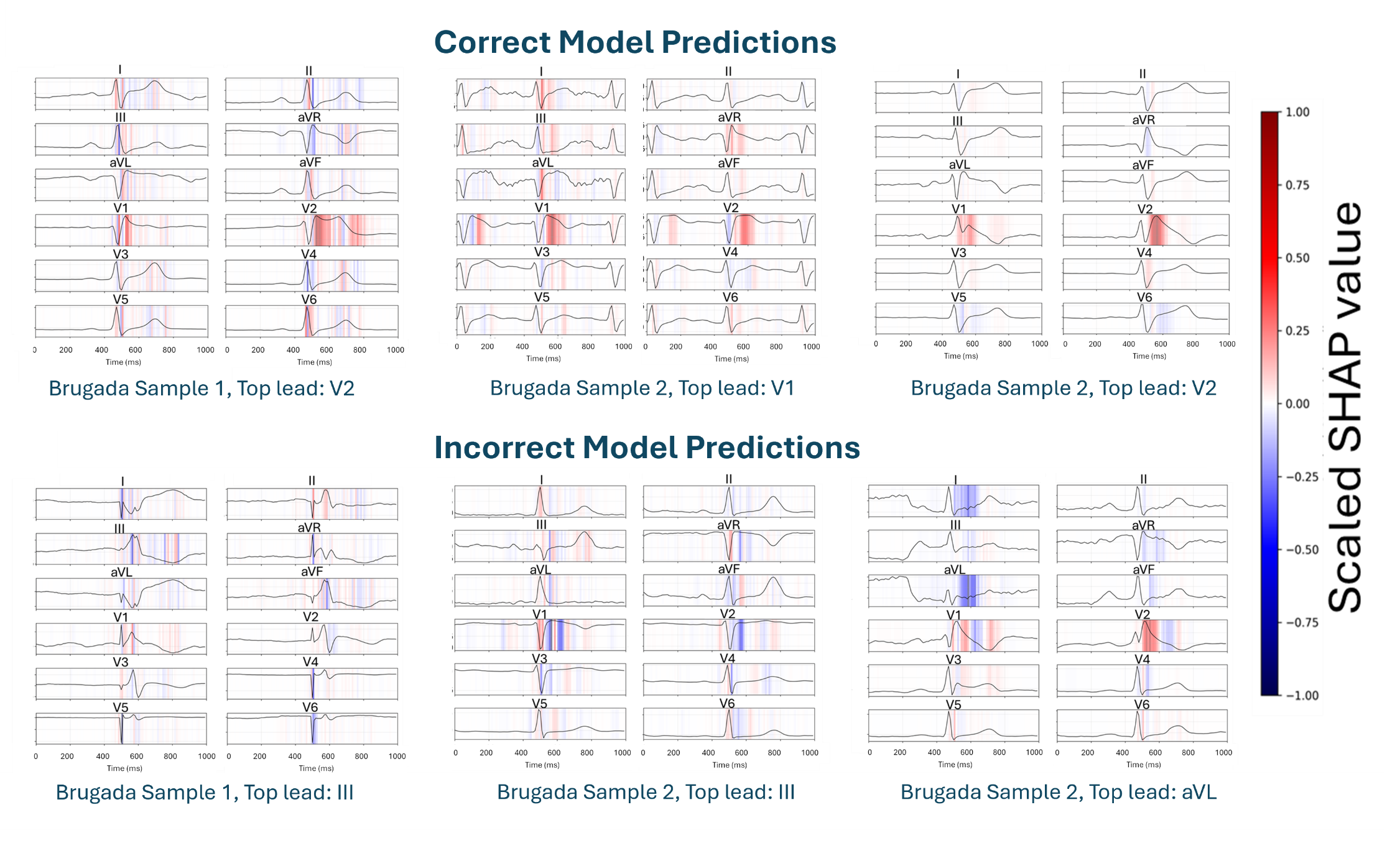
The top row shows three Brugada cases that were correctly classified by the model, and the bottom row shows three Brugada cases that were misclassified as not Brugada. For each case, all 12 leads are displayed with a time-resolved SHAP heat overlay (colour bar at the right). Red indicates time points/segments that increase the model’s predicted probability of Brugada, whereas blue indicates segments that decrease it (SHAP values scaled to -1 to 1 for visualisation).

**Fig D. t-SNE visualisation comparing real and SMOTE-generated Brugada ECGs under different data availability conditions.**

| 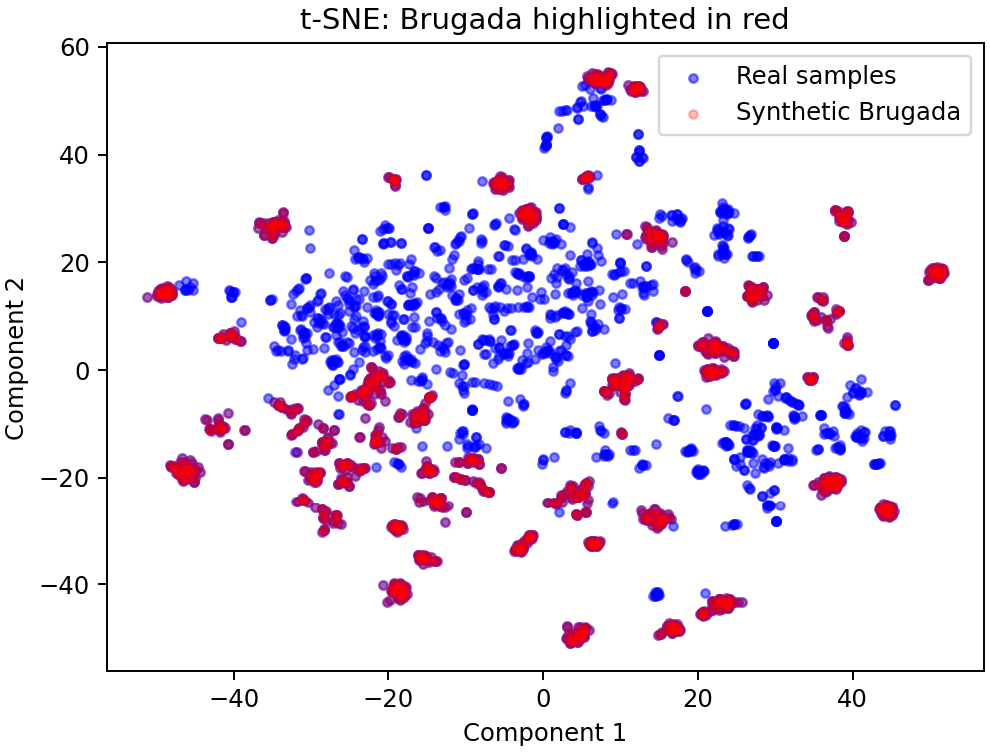 | 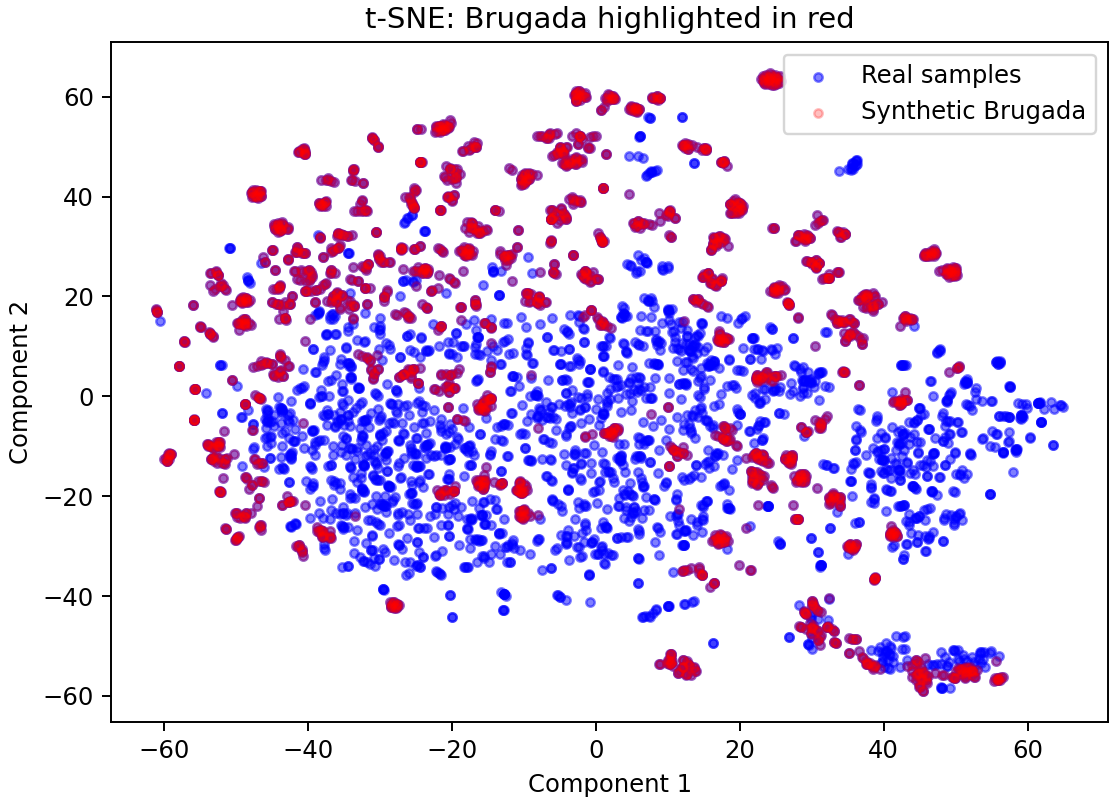 |
| --- | --- |
| 25%-75% split | 80%-20% split |

The left panel shows the 25%-75% train-test split with limited training data, while the right panel shows the 80%-20% split with more abundant training data. Real Brugada ECGs are shown as blue dots, and SMOTE-generated synthetic Brugada ECGs are shown as red dots. The clustering of synthetic samples closely with real samples demonstrates morphological plausibility but reveals limited diversity in the synthetic data generation, with this pattern consistent across both data scarcity conditions. *Note: t-SNE visualizations are provided for illustrative purposes to show qualitative clustering patterns and should not be used for quantitative inference regarding the underlying global data structure or distances.*

# **Synthetic ECG generation using a variational autoencoder**

To explore deep generative modelling as a potential strategy for minority class augmentation, we trained a convolutional Variational Autoencoder (VAE) with skip connections, inspired by U-Net architectures. The VAE was trained on the entire training set, comprising a mixture of normal, RBBB, and Brugada beats from the baseline 80%-20% dataset split.

The encoder comprised four 1D convolutional layers with increasing filter sizes, each followed by batch normalisation and ReLU activation. A global average pooling layer projected the output to a 512-dimensional latent space via dense layers, yielding the mean and log-variance of the latent distribution. Latent vectors were sampled using the reparameterization trick to enable backpropagation through the stochastic layer.

The decoder mirrored the encoder with three upsampling blocks, each incorporating resized skip connections from corresponding encoder layers. The final output reconstructed a 12-lead, 1-second ECG (shape: 100×12), cropped to remove edge artefacts.

The model was trained using a standard VAE loss combining mean squared reconstruction error and KL divergence, optimised with Adam (1e^-4^) over 4000 epochs. Training used all available data (batch size 128), with 20% held out for validation. During cross-fold validation, to synthesise Brugada samples, latent vectors were extracted from Brugada-labelled ECGs in each fold. A multivariate Gaussian was fitted to these embeddings, from which new samples were drawn. To reconstruct signals, real skip connections were tiled and reused to preserve temporal structure.

We evaluated reconstruction fidelity using the normalised L2 error metric between original and reconstructed training signals. The average reconstruction error across the training set was 2.5 ± 0.5%, with a higher variability on the test set (5.2 ± 3.3%). While the generated ECGs exhibited plausible high-level morphology, visual inspection revealed limited inter-lead coherence, shown in **Fig E.**

**Fig E. Representative synthetic ECG samples generated using a variational autoencoder (VAE).**

| **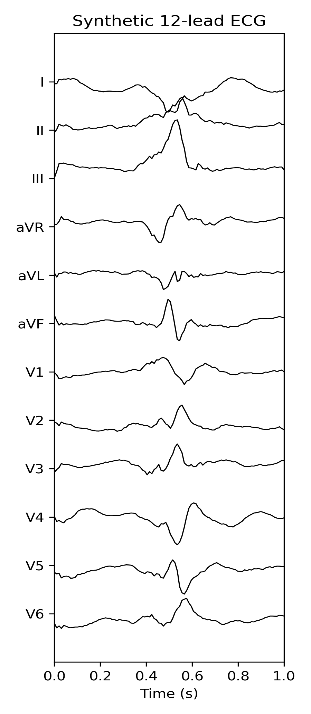** | **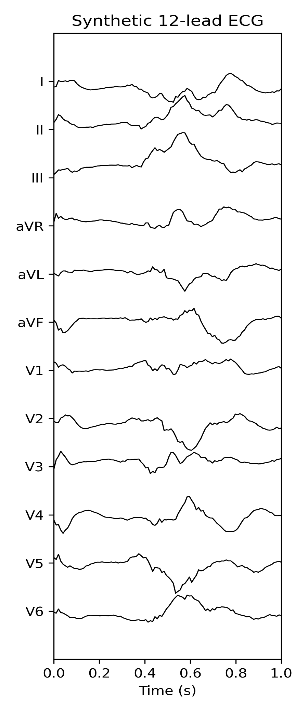** | **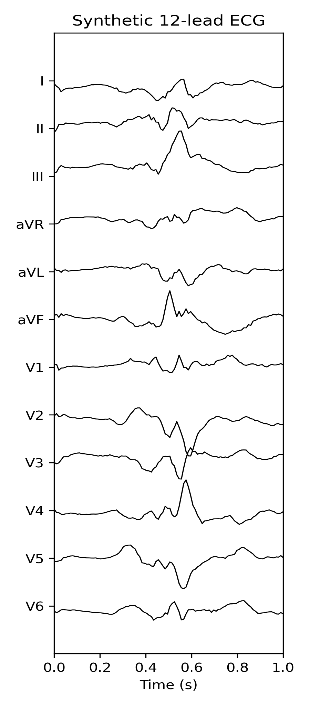** | **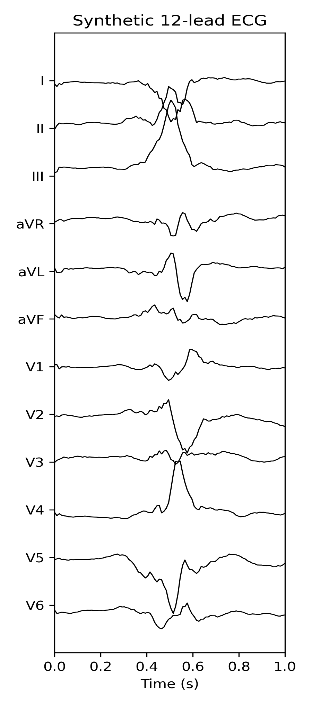** | One synthetic Brugada sample is shown per fold (8 folds total). While the generated signals exhibit recognizable morphological structures such as P waves, QRS complexes, and T waves. The overall clinical fidelity varies across samples and was not consistent. |
| --- | --- | --- | --- | --- |
| **Fold 1** | **Fold 2** | **Fold 3** | **Fold 4** |  |
| **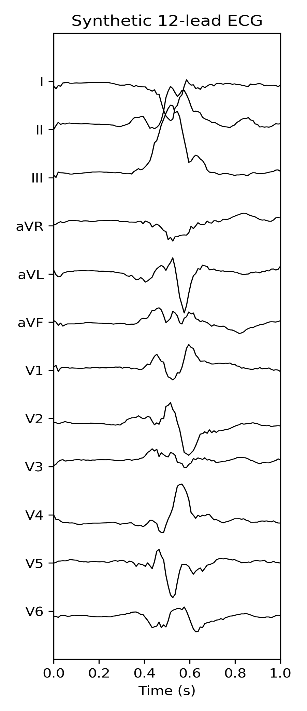** | **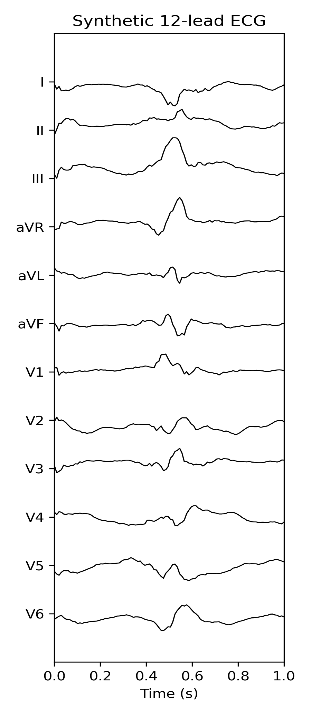** | **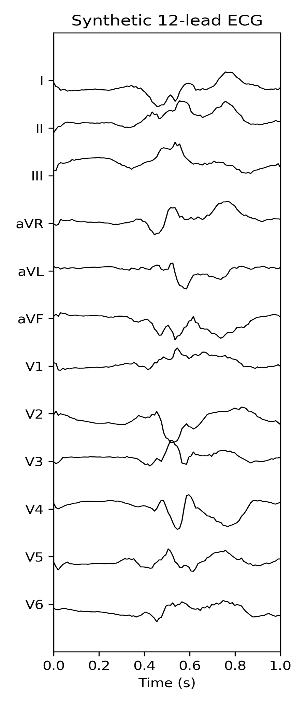** | **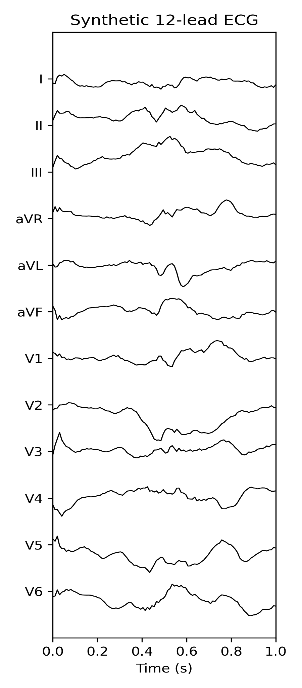** |  |
| **Fold 5** | **Fold 6** | **Fold 7** | **Fold 8** |  |
